# *E. coli* NF73-1 Isolated From NASH Patients Aggravates NAFLD in Mice by Translocating Into the Liver and Stimulating M1 Polarization

**DOI:** 10.3389/fcimb.2020.535940

**Published:** 2020-12-11

**Authors:** Yifan Zhang, Weiwei Jiang, Jun Xu, Na Wu, Yang Wang, Tianyu Lin, Yun Liu, Yulan Liu

**Affiliations:** ^1^ Department of Gastroenterology, Peking University People's Hospital, Beijing, China; ^2^ Clinical Center of Immune-Mediated Digestive Diseases, Peking University People's Hospital, Beijing, China; ^3^ Department of Gastroenterology, The First Affiliated Hospital of Shandong First Medical University, Jinan, China; ^4^ Institute of Clinical Molecular Biology & Central Laboratory, Peking University People’s Hospital, Beijing, China

**Keywords:** bacteria strain, non-alcoholic steatohepatitis, triglyceride accumulation, macrophage, hepatic inflammation

## Abstract

**Objective:**

The gut microbiota is associated with nonalcoholic fatty liver disease (NAFLD). We isolated the *Escherichia coli* strain NF73-1 from the intestines of a NASH patient and then investigated its effect and underlying mechanism.

**Methods:**

16S ribosomal RNA (16S rRNA) amplicon sequencing was used to detect bacterial profiles in healthy controls, NAFLD patients and NASH patients. Highly enriched *E. coli* strains were cultured and isolated from NASH patients. Whole-genome sequencing and comparative genomics were performed to investigate gene expression. Depending on the diet, male C57BL/6J mice were further grouped in normal diet (ND) and high-fat diet (HFD) groups. To avoid disturbing the bacterial microbiota, some of the ND and HFD mice were grouped as “bacteria-depleted” mice and treated with a cocktail of broad-spectrum antibiotic complex (ABX) from the 8^th^ to 10^th^ week. Then, *E. coli* NF73-1, the bacterial strain isolated from NASH patients, was administered transgastrically for 6 weeks to investigate its effect and mechanism in the pathogenic progression of NAFLD.

**Results:**

The relative abundance of *Escherichia* increased significantly in the mucosa of NAFLD patients, especially NASH patients. The results from whole-genome sequencing and comparative genomics showed a specific gene expression profile in *E. coli* strain NF73-1, which was isolated from the intestinal mucosa of NASH patients. *E. coli* NF73-1 accelerates NAFLD independently. Only in the HFD-NF73-1 and HFD-ABX-NF73-1 groups were EGFP-labeled *E. coli* NF73-1 detected in the liver and intestine. Subsequently, translocation of *E. coli* NF73-1 into the liver led to an increase in hepatic M1 macrophages via the TLR2/NLRP3 pathway. Hepatic M1 macrophages induced by *E. coli* NF73-1 activated mTOR-S6K1-SREBP-1/PPAR-α signaling, causing a metabolic switch from triglyceride oxidation toward triglyceride synthesis in NAFLD mice.

**Conclusions:**

*E. coli* NF73-1 is a critical trigger in the progression of NAFLD. *E. coli* NF73-1 might be a specific strain for NAFLD patients.

## Introduction

The gut-liver axis is the passageway by which the gut microbiota and gut-derived hepatotoxic bacterial products can easily enter the liver (Wiest et al., 2017; Chu et al., 2019). Interplay between the gut microbiota and liver injury through the gut-liver axis is known to play a pivotal role in the development and progression of nonalcoholic fatty liver disease (NAFLD) (Schnabl and Brenner, 2014; Chu et al., 2019). Emerging evidence has demonstrated that the gut microbial profile in NAFLD and/or nonalcoholic steatohepatitis (NASH) patients is strikingly different from that of healthy subjects (Safari and Gerard, 2019; Kim et al., 2019; Kolodziejczyk et al., 2019). NAFLD patients generally exhibit decreased microbial diversity compared with healthy controls (Del Chierico et al., 2017). In addition, *Proteobacteria*, which includes *Escherichia*, is the most common differentially abundant taxon between obese patients with NASH and those without NASH (Boursier et al., 2016). Many studies have found that germ-free or antibiotic-treated (bacteria-depleted) mice exhibited less lipid accumulation and inflammation than conventional mice fed a high-fat diet (HFD), which highlights the pivotal role of the gut microbiota in the natural progression of NAFLD (Backhed et al., 2004; Brandt et al., 2017; Wang et al., 2018). Moreover, after being administered the microbiota of NASH patients orally by gavage, HFD-fed germ-free mice showed an exacerbated NASH phenotype, manifesting as increased liver steatosis and inflammation (Chiu et al., 2017). The gut microbiota, in brief, has been indicated to play a critical role in the development of NAFLD.

Until now, most studies have determined the bacterial composition of intestinal feces at the genus level but have failed to identify bacteria at the species or strain level. In previous studies, a common finding in NAFLD patients was an increase in *Escherichia* at the genus level (Zhu et al., 2013; Mouzaki et al., 2013; Jiao et al., 2018). Furthermore, it has been reported that *Escherichia* is a unique genus that is significantly elevated in NASH patients compared with healthy controls and obese patients (Li et al., 2017). Another study confirmed that *Escherichia* was the predominant bacterium in NAFLD patients with small intestinal bacterial overgrowth (SIBO) (Kapil et al., 2016). Additionally, based on our previous research, a higher abundance of fecal *Escherichia* was observed in the Chinese population with NAFLD than in healthy subjects; of note, the abundance of *Escherichia* increased gradually in the progression of NAFLD to NASH (Jiang et al., 2015). These facts suggest that *Escherichia* can be a risk factor in the disease progression from NAFLD to NASH.

Of note, several studies have shown that multiple bacterial genera are associated with various diseases, and different strains of the same genus might have different pathogenic mechanisms (Kostic et al., 2013; Tsoi et al., 2017; Yu et al., 2017). *Escherichia coli* is an opportunistic pathogen that exists in NAFLD patients and healthy controls and is generally considered safe under normal health conditions. Increased bacterial colonization by *E. coli* or increased expression of virulence-related genes of *E. coli* may be inherently pathogenic (Kaper et al., 2004; Zargar et al., 2015). In addition, translocation of gut microbes was reported to trigger the progression of liver disease. *Enterococcus gallinarum* was reported to translocate into the liver in autoimmune hepatitis (AIH) (Manfredo Vieira et al., 2018). Previous studies have also shown that NAFLD is influenced by pathological bacterial translocation (PBT) from the gut (Wu et al., 2018). Therefore, we became interested in the effect and mechanism of gut microbes, especially *E. coli*, in the pathogenic progression of NAFLD.

Few studies have focused on the mucosal microbiota, especially bacterial strains, from the intestinal mucosa of clinical patients. In this study, to determine the relative bacterial abundance in mucosal samples at the genus level among healthy, NAFLD and NASH groups. We performed 16S ribosomal RNA (16S rRNA) amplicon sequencing. The data showed that, compared with the healthy group, the relative abundance of *E. coli* was increased in the NASH and NAFLD groups, especially in the NASH group, which was consistent with the changes in stool samples. These results further confirmed the accumulation of *E. coli* in the pathogenic progression from NAFLD to NASH. Bacterial cultivation, isolation and full-length 16S rRNA sequencing were performed to cluster the strains of *E. coli* enriched in NASH patients. We named the most abundant *E. coli* strain *E. coli* NF73-1 (authorized patent in China: No. 201610591293.4). In addition to bacterial abundance, the expression of virulence-related genes was also reported to be inherently pathogenic (Kaper et al., 2004; Zargar et al., 2015); thus, we performed whole-genome sequencing of *E. coli* NF73-1. The data showed enrichment of adherence genes and pathogenic genes, such as *fimH* (Chen et al., 2009), *ybt* (Ellermann et al., 2019), etc. in the genome of *E. coli* NF73-1(**Table S1**). Based on its highest abundance in NASH patients and its genetic pattern, we inferred that *E. coli* NF73-1 played a pivotal role in the progression of NAFLD.

The overall aim of the current study was to investigate the effect of *E. coli* NF73-1 in the progression of NAFLD, especially in liver inflammation and lipid metabolism.

## Materials and Methods

### Patients and Mucosa Collection

This cross-sectional study was approved by the Conjoint Health Research Ethics Board of Peking University People’s Hospital, and informed consent forms were signed by all the subjects prior to participation in this study. The diagnosis of NAFLD was established according to the Gastroenterology Organization Guidelines (Chalasani et al., 2012).

A total of 19 NAFLD patients were recruited at Peking University People’s Hospital. All patients were confirmed to exhibit NAFLD based on either imaging or histological evidence. Additionally, 10 healthy subjects were invited to participate as controls (**Table 1**). The 29 subjects agreed to undergo gastroscopy for physical examination and signed the appropriate informed consent form. In all cases, the mucosa samples were collected prior to endoscopy. On the day prior to the gastroscopic procedure, a blood sample was collected to measure the metabolic and hepatic parameters.

**Table 1:** Clinical and biochemical features of patients with NAFLD, NASH patients and the control group.

Variables	Control subjects	NAFLD	NASH
Number of subjects	10	10	9
Female/male (n)	6/4	4/6	3/6
Age, years	55.20±2.77	55.90±3.40	52.33±2.88
BMI, kg/m^2^	21.62±0.77	27.95±0.71***	26.08±1.09**
ALT(U/L)	14.96±1.75	24.48±3.76	64.87±7.39***^###^
AST(U/L)	20.11±1.15	21.56±1.26	37.52±3.51***^###^
TG (mmol/L)	0.98±0.14	2.10±0.36*	2.81±0.44***
CHO (mmol/L)	4.74±0.38	5.43±0.26	4.77±0.53

*Healthy vs NASH, NAFLD; ^###^NAFLD vs NASH; *p <0.05, **p<0.01, ***p<0.001, ^###^p<0.001 (unpaired t test).

### Mucosal Bacterial 16S rRNA Sequencing and Analysis

Microbial genomic DNA was extracted according to the manufacturer’s instructions, with minor modifications (Qiagen, Hilden, Germany). After DNA extraction, bacterial *16S rDNA* was amplified. Briefly, the V3–V4 region of *16S rDNA* was amplified using paired primers (357F/806R). After library preparation and quantification (Thermo Scientific, USA), paired-end sequencing (2 × 150 bp) was performed on an Illumina HiSeq 2500 sequencer at the Center for Molecular Immunology of Chinese Academy of Sciences (Beijing, China). In addition, raw fastq files were quality-filtered using the fastq_quality_filter (-p 90 -q 25 -Q33) in FASTX-Toolkit (v.0.0.14) and merged by Fast Length Adjustment of SHort reads (FLASH) (Caporaso et al., 2010; Edgar et al., 2011; Magoc and Salzberg, 2011). The OTUs were aligned utilizing the UCLUST algorithm with 97% identity and taxonomically classified using the SILVA database, v128, released on 29/09/2016. Additionally, the relative abundances of the various phyla, classes, orders, families, and genera in each sample were computed and compared among all groups. The detailed methods have been described in our previous studies (Xu et al., 2018; Jun et al., 2019).

#### Identification and classification of *E. coli* in NAFLD/NASH

Based on the 16S rRNA sequencing analysis, the significantly enriched *E. coli* were chosen for further study. First, *E. coli*-specific culturing was performed to pick pathogenic strains. Approximately 100 strains of *E. coli* were cultured and subjected to full-length 16S rRNA sequencing. The data showed 99% similarity with *E. coli* in the NCBI database. To analyze the evolutionary relationship among the strains, a phylogenetic tree was established with kSNP3 software (Gardner et al., 2015) using the neighbor-joining (NJ) method. Based on the similarity of genomic sequences, three types of *E. coli* subspecies were identified, i.e., NF73, NF4 and NF23.

### *E. coli*-specific Comparative Genomic Analysis

*E. coli* NF73-1 was used for full-length genomic sequencing and further comparative genomic analysis. The genome of *E. coli* NF73-1 was sequenced using an Illumina HiSeq 4000 system (Illumina, San Diego, CA, USA) at the Beijing Genomics Institute (Shenzhen, China). The sequenced reads were assembled using SOAPdenovo v1.05 software.

For phylogenetic analysis based on full-length 16S rRNA, the evolutionary history was inferred by using the maximum likelihood method based on the Tamura-Nei model. Evolutionary analyses were conducted in MEGA7. A phylogenetic tree based on the whole-genome sequence was constructed by TreeBeST using the NJ method.

Gene prediction was performed on the *E. coli* NF73-1 genome assembly by glimmer3 with hidden Markov models. tRNA, rRNA and sRNA recognition was conducted with tRNAscan-SE (Lowe and Eddy, 1997), RNAmmer, and the Rfam database. Tandem repeat annotation was obtained using the Tandem Repeat Finder (http://tandem.bu.edu/trf/trf.html), and the minisatellite DNA and microsatellite DNA were selected based on the number and length of repeat units. The Genomic Island Suite of Tools (GIST) was used for genomic island analysis (http://www5.esu.edu/cpsc/bioinfo/software/GIST/) with the IslandPath-DIOMB, SIGI-HMM, and IslandPicker methods. Prophage regions were predicted using the PHAge Search Tool (PHAST) web server (http://phast.wishartlab.com/), and CRISPR identification was performed using CRISPR Finder. The best hit was abstracted using the BLAST alignment tool for functional annotation. Database, namely Kyoto Encyclopedia of Genes and Genomes (KEGG) were used for general function annotation.

According to the dispensable gene in different strains, a heat map showed the cluster analysis among *E.coli* strains. A detailed description of genomes can be found in **Table S1**. Diamond (V0.9.18.119) software was used to compare the sequences of 2 strains in the KEGG database, and the annotation information for KEGG was obtained (Kanehisa et al., 2014). The key genes were selected to compare and display the heatmap.

### Animal Experiments

Six-week-old C57BL/6J specific-pathogen-free (SPF) male mice were purchased from the Vital River facility (Beijing, China). Animals were housed with free access to food and sterile drinking water in a temperature-controlled room (21°C ± 2°C) under a 12-h dark-light cycle in a SPF facility. Male C57BL/6J SPF mice were divided into a conventional group and a flora-deficient group. Then, both groups were fed either a normal diet (ND) or an HFD (45% kcal fat; Mediscience Ltd, China) (**Table 6**) for 16 weeks. Depletion of the gut microbiota was performed as previously described, with slight modifications (Tsoi et al., 2017; Schneider et al., 2019). After 8 weeks, the flora-deficient group was treated with a cocktail of broad-spectrum antibiotic complex (ABX, 1 g/L ampicillin, 0.5 g/L vancomycin, 1 g/L neomycin sulfate, and 1 g/L metronidazole) for 2 weeks to eliminate intestinal bacteria. After week 10, all groups were further treated intragastrically with 1 × 10^8^ colony-forming units (CFU) of *E. coli* NF73-1 (HFD-NF73-1 group, ND-NF73-1 group, HFD-ABX-NF73-1 group and ND-ABX-NF73-1 group), Luria-Bertani (LB) medium (HFD-LB group, ND-LB group, HFD-ABX-LB group and ND-ABX-LB group), or 1 × 10^8^ CFU of the control bacterium *E. coli* MG1655 (HFD-MG1655 group, ND-MG1655 group, HFD-ABX-MG1655 group and ND-ABX-MG1655 group) every day.

For depletion of liver macrophages, we injected flora-deficient HFD mice with liposome-encapsulated clodronate (CLOD) (0.1 mL per 10 grams, F7010C-AC, Formumax, USA) intravenously once for the first week and then twice per week (0.05 mL per 10 grams) for the following five weeks. Liposome-encapsulated phosphate-buffered saline (PBS) was used as a control (CLOD CON).

At the time indicated, animals fasted for 12 h were anesthetized, and whole blood was withdrawn by cardiac puncture. Serum and tissues were collected, including the liver and intestine.

### Stool DNA Extraction

For optimal isolation of bacterial DNA, mucosal biopsies were disrupted by bead-beating after digestion with an enzymatic cocktail of mutanolysin and lysozyme (Sigma-Aldrich, USA). Then, extraction and purification were performed using the QIAamp DNA Mini Kit. Stool DNA was quantified using a Nanodrop-1000 (Thermo Scientific, USA).

#### Serum biochemical measurements

Serum was collected from blood and assayed for levels of alanine aminotransferase (ALT), aspartate aminotransferase (AST), total triglyceride (TG) and cholesterol (TC) using an automatic biochemical detector (Labospect 008 AS, Japan).

### Oil Red O Staining and H&E Staining

For Oil Red O staining, optimal cutting temperature (OCT)-embedded frozen tissue was sectioned at 5 to 7 μm. After washing with PBS, dried slides were subsequently incubated with 100% propylene glycol and Oil Red O solution (ab150678; Sigma, USA). Stained slides were differentiated with 85% propylene glycol solution and distilled water. Then, stained slides were incubated in hematoxylin and mounted with glycerin.

For hematoxylin and eosin (H&E) staining, liver tissue was fixed in 10% neutral buffered formalin (Sericebio; China). Paraffin-embedded liver tissues were used. The NAFLD activity score was assessed by two blinded investigators.

### Isolation of MNCs in the Liver

Livers were perfused with PBS via the portal vein to eliminate the effects of lymphocytes in blood. In brief, mouse livers were homogenized and filtered through a 70-μm nylon cell strainer (BD Bioscience, San Jose, CA, USA). Liver mononuclear cells (MNCs) were separated from nonparenchymal cells using density separation in a Percoll gradient (17089101, GE, USA). Percoll is silica sol with nondialyzable polyvinylpyrrolidone coating, the density of which is 1.13±0.005 g/mL. We prepared 80% Percoll solution: Mix 4 ml of Percoll separation solution with 36 ml of sterile 10× PBS and 10 ml of sterile 1× PBS. We prepared 40% Percoll solution by mixing 2 ml of Percoll separation solution with 18 mL of sterile 10× PBS and 30 ml of sterile 1× PBS. The density of 40% Percoll is less than 1.050 g/mL. The density of mononuclear cells is 1.050 to 1.095 g/mL. The density of 80% Percoll is greater than 1.095 g/mL. After removing debris, hepatic nonparenchymal cells were collected and suspended in 40% Percoll. The cell suspension was gently overlaid onto 80% Percoll and centrifuged at 4°C for 25 min at 3000 r.p.m. Liver mononuclear cells (MNCs) were collected from the interface. Then, the MNCs were resuspended in fresh PBS after red blood cell lysis (Biolegend, USA). In brief, FcγR was first blocked with anti-CD16/CD32 antibody (TruStain fcX, Biolegend) at 4°C for 10 min. All resuspended cells were further sorted by a Gallios instrument (Beckman, USA) using the following antibodies (Biolegend, USA): AF700-conjugated anti-mouse CD45, APC-Cy7 tagged anti-mouse F4/80 and PE-Cy7-conjugated anti-mouse CD11b, BV-570-conjugated anti-mouse Ly-6G, BV421 tagged anti-mouse CD86, and FITC conjugated anti-mouse CD206. Natural killer (NK, NK1.1^+^CD3^−^) cells, natural killer T (NKT, NK1.1^+^CD3^+^) cells, neutrophils (CD11b^+^Ly6G^+^), CD4 T cells (CD3^+^CD4^+^ CD8^-^), CD8 T cells (CD3^+^CD8^+^CD4^-^), macrophages (CD11b^+^F4/80^+^) and B (CD3^-^B220^+^) cells were also analyzed through pseudocolor analysis plots. Stained cells were read with a Gallios instrument. The gating strategy is illustrated in **Figures S3C, D**. The results were analyzed using Beckman software.

### Immunochemical Staining

Liver tissues were removed at sacrifice and then fixed in formalin, embedded in paraffin, and prepared for histology. The liver tissue slides were blocked with 10% goat serum for 30 min, followed by incubation for 1 h with anti‐F4/80 antibody (1:200; ab16911, Abcam, China) and anti‐pS6 antibody (1:200; 2211, CST, USA) at 4°C overnight. After incubation with a poly‐peroxidase‐conjugated goat anti‐rat IgG or goat anti‐rabbit IgG (Zhongshan Golden Bridge, China) at 37°C for 30 min, the slides were counterstained with 3,3′‐diaminobenzidine (DAB) and hematoxylin and eventually mounted with glycerin.

### RT-qPCR

Total RNA from the liver and macrophages was isolated TRIzol reagent (15596; Thermo Fisher Scientific) and reverse transcribed to complementary DNA by the qScript Reverse Transcriptase Kit (Thermo Fisher Scientific, USA). Gene expression profiles were quantified by quantitative PCR using the ABI 7500 Real-Time PCR System (ABI) with SYBR FAST qPCR Kits (Transgen, China). mRNA amounts were calculated by the comparative cycle threshold method (ΔΔCt method) with normalization to actin mRNA. Primers were purchased from Sangon Company (Shanghai) and are listed in **Table 2**.

**Table 2:** Primers.

Genes	Forward(5’-3’)	Reverse(5’-3’)
β-actin	TGTCCACCTTCCAGCAGATGT	AGCTCAGTAACAGTCCGCCTAG
IL-6	TAGTCCTTCCTACCCCAATTTCC	TTGGTCCTTAGCCACTCCTTC
IL-1β	GAAATGCCACCTTTTGACAGTG	TGGATGCTCTCATCAGGACAG
TNF-α	CCCTCACACTCAGATCATCTTCT	GCTACGACGTGGGCTACAG
iNOS	GTTCTCAGCCCAACAATACAAGA	GTGGACGGGTCGATGTCAC
EGFP	GGGCTGGCAAGCCACGTTTGGTG	CCGGGAGCTGCATGTGTCAGAGG
Arg	CTCCAAGCCAAAGTCCTTAGAG	AGGAGCTCATTAGGGACATC
Mrc2	TACAGCTCCACGCTATGGAT	CACTCTCCCAGTTGAGGTACT
IL-10	GTTACTTGGGTTGCCAAG	GATCATCATGTATGCTTC
TLR2	GCAAACGCTGTTCTGCTCAG	AGGCGTCTCCCTCTATTGTATT
TLR4	ATGGCATGGCTTACACCACC	GAGGCCAATTTTGTCTCCACA
TLR7	ATGTGGACACGGAAGAGACAA	GGTAAGGGTAAGATTGGTGGTG
TLR9	ATGGTTCTCCGTCGAAGGACT	GAGGCTTCAGCTCACAGGG
P50	ATGGCAGACGATGATCCCTAC	TGTTGACAGTGGTATTTCTGGTG
P65	AGGCTTCTGGGCCTTATGTG	TGCTTCTCTCGCCAGGAATAC
MAPK	GCGGGATTCTACCGGCAAG	GAGCAGACTGAGCCGTAGG
ERK	GGTTGTTCCCAAATGCTGACT	CAACTTCAATCCTCTTGTGAGGG
NLRP3	ATTACCCGCCCGAGAAAGG	TCGCAGCAAAGATCCACACAG
CASPASE-1	ACAAGGCACGGGACCTATG	TCCCAGTCAGTCCTGGAAATG

### Liver TG and TC Levels

Hepatic lipids were extracted from 50 mg of liver tissue and assayed using a triglyceride assay kit and a cholesterol assay kit (E1013, E1015, Applygen Technologies, China) according to the manufacturer’s recommended protocols. Protein concentrations were quantified by using a Pierce BCA Protein Assay Kit (23227, Thermo, USA).

### Cell Coculture and Oil Red O Staining (RAW 264.7, L02, H2.35, *E. coli* NF73-1)

RAW 264.7 cells (obtained from ATCC and free from mycoplasma) were stimulated with 1 mM palmitate and oleic acid conjugated to fatty acid-free bovine serum albumin (BSA) (A2000, Applygen Technologies, China) or only fatty acid-free BSA for 1 h and then cultured with or without NF73-1. Then, surface immunofluorescence antibody staining was performed quickly for identification of macrophages. L02 and H2.35 (obtained from ATCC and free from mycoplasma) were stimulated with 1 mM palmitate and oleic acid conjugated with fatty acid-free BSA or only fatty acid-free BSA for 20 h and then cocultured with or without NF73-1 for 4 h. After washing with PBS, the cells were incubated with 4% neutral buffered formalin and Oil Red O solution (ab150678; Sigma, USA). Stained slides were washed with 85% propylene glycol solution and distilled water and then mounted with glycerin.

### Bacterial Culture

*E. coli* NF73-1 and *E. coli* MG1655 were transformed with plasmid CV129-EGFP to induce the expression of enhanced green fluorescent protein (EGFP) and β-lactamase, conferring resistance to ampicillin, to follow bacterial translocation from the intestine. *E. coli* EGFP*-*NF73-1 and *E. coli* EGFP*-*MG1655 were cultured in LB under aerobic conditions. Primers of EGFP were purchased from Sangon Company (Shanghai) and are listed in **Table 2**.

All experiments were performed under strict sterile conditions. At the time indicated, livers (100 mg each) were removed aseptically, homogenized, plated on ampicillin-supplemented Luria Broth plates and cultured at 37°C overnight. The CFUs were then counted to quantify bacteria.

### Immunofluorescence Staining and Confocal Imaging Assay

Liver and intestinal tissues were fixed in OCT (4583, Sakura Finetek, USA) and cut at 5-µm thickness using a Leica freezing slicer. The tissue slides were blocked with 10% goat serum for 30 min and incubated with 4′,6′-diamidino-2-2phenylindole (DAPI) (ZLI-9557, Zhongshan Golden Bridge, China) to stain the nuclei. The slides were observed using a confocal microscope (Nikon A1R, Tokyo, Japan).

### Western Blotting

Total protein samples were isolated from liver tissue and cells cultured using RIPA lysis buffer (Thermo Fisher Scientific, USA) containing protease and phosphatase inhibitors (Roche, Basel, Switzerland). Samples were separated by 10% SDS-polyacrylamide gel electrophoresis and transferred onto polyvinylidene difluoride membranes (Millipore, CA). The membranes were blocked in 5% milk and incubated with primary antibodies against mTOR (2983T), p-mTOR (5536T), p70S6 (9234), S6 (2217), pS6 (2211), TLR2 (13744), pNF-κBp65 (3033), NF-κBp65 (4764), NF-κBp50 (13586) and β-actin (4970) (CST, USA) at 1:1000 in TBST. Antibodies against PPAR-α (ab24509), SREBP-1 (ab28481), FASN (ab22759), ACC1 (ab45174), S6K1 (ab32529) and Caspase-1 (ab1872) were obtained from Abcam (Cambridge, USA). The intensity of the bands was analyzed by scanning densitometry and quantified by NIH Image J software. For protein expression, the specific band intensity was quantified, normalized to those of β-actin, and presented as a value relative to the control.

### Coculture of *E. coli* NF73-1, RAW264.7, and Hepatocytes

RAW264.7 macrophages (2 × 10^3^ cells/ml) were cultured in DMEM with oleic acid and palmitic acid in the upper chamber, and L02 and H2.35 (2 × 10^5^ cells/ml) were cocultured in the lower chamber of a 6-well Transwell plate (3412; Corning, USA) for 20 h; the chambers were separated by a 0.4-µm track-etched membrane. Then, *E. coli* NF73-1 or rapamycin was added to the coculture system. After 4 h of cultivation, hepatocytes were collected for Western blot analysis.

In another experiment, after 4 h of cultivation with *E. coli* NF73-1, RAW264.7 cells were collected and labeled with APC-Cy7-F4/80, CD11b-PerCP, BV421-CD86, and CD206-FITC antibodies and detected using a Gallios (Beckman, USA) flow cytometer. Moreover, RAW264.7 cells were harvested for RNA extraction and Western blot analysis. The gating strategy is illustrated in **Figure S3E**. The data were analyzed using Beckman software.

### Statistical Analysis

Statistical analysis was performed using GraphPad Prism (version 5.01, San Diego, CA, USA). The results are shown as the mean ± S.E.M. (standard error mean). The results were analyzed by one-way analysis of variance between multiple groups when appropriate, and by a two-tailed Student’s *t* test for comparison of two groups. P < 0.05 was considered statistically significant.

## Results

### Isolation and Identification of *E. coli* Strains From the Intestinal Mucosa of NASH Patients

**Figure 1:**
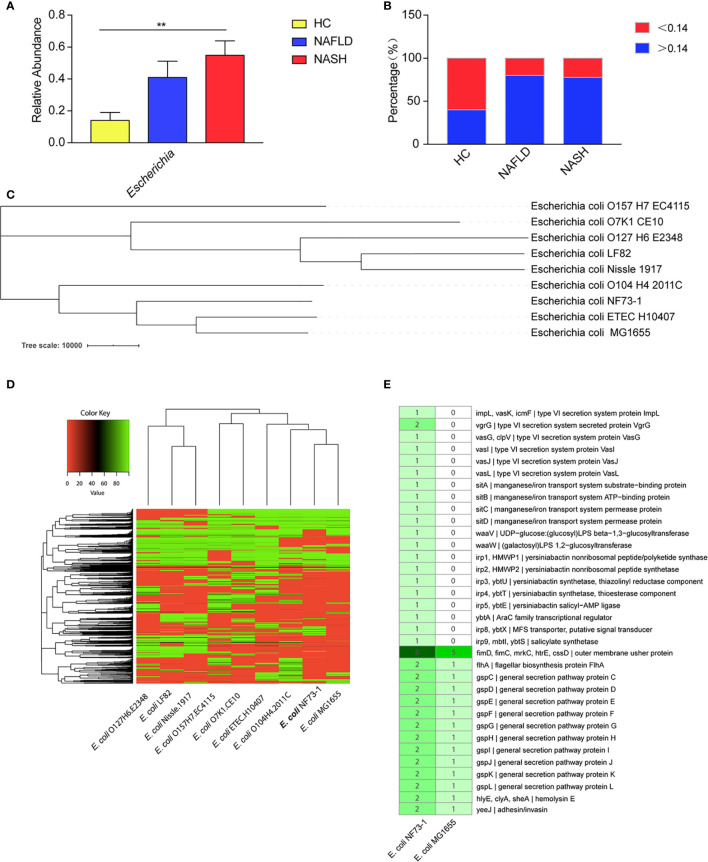
*E. coli*–specific comparative genomic analysis. **(A)** Comparison of the *Escherichia* abundance at the genus level among NASH and NAFLD patients and healthy subjects. **(B)** High-abundance (>0.14) *Escherichia* colonized percentage of the healthy controls, NAFLD and NASH patients. **(C)** A phylogenetic tree was constructed by TreeBeST using the neighbor-joining (NJ) method. **(D)** Cluster heat map of 9 *E. coli* genomes. The cluster tree of dispensable genes is represented on the Y-axis and the *E. coli* genomes are listed on the X axis (*E. coli*. O127H6.E2348, *E. coli*. LF82, *E. coli*. Nissle.1917, *E. coli.* O157H7.EC4115, *E. coli.* O7K1.CE10, E*. coli*. ETEC.H10407, *E. coli*. O104H4.2011C, *E. coli*.MG1655 and *E. coli.* NF73-1). The color key representing the degree of similarities and differences of the genomes based on dispensable genes. A detailed description of genomes can be found in **Table S1**. **(E)** Comparative genomics of *E. coli* NF73-1 and *E. coli* MG1655 in KEGG general functional annotations. Gene names are represented on Y-axis and the *E. coli* genomes are listed on the X-axis. The color key representing the degree of differences of the genomes based on the presence or absence of genes. Detail description of genomes can be found in **Table S3**. Data are calculated as the mean ± SEM, n = 9–10 per group. **p <0.01 (one-way analysis of variance between multiple groups).

To explore the key bacterial strains involved in the pathogenesis of NAFLD, especially mucosa-adherent bacterial strains, we analyzed the abundance of *Escherichia* in mucosal samples from NAFLD patients recruited at Peking University People’s Hospital. An increased abundance of bacteria belonging to *Escherichia* within the phylum *Proteobacteria* was observed in the microbiota from the NASH and NAFLD groups compared with the healthy group. Seven genera, namely *Bacteroides*, *Faecalibacterium*, *Prevotella*, *Blautia*, *Lachnospiracea*, *Clostridium*, and *Roseburia*, were less abundant in both the NAFLD and NASH patients than the healthy subjects (**Figure S1A**). In addition, the relative abundance of *Escherichia* in the NASH group was higher than that in the NAFLD group (**Figure 1A**). The changes in *Escherichia* in the mucosal flora of these groups were consistent with the fecal flora in our previous research. In our data, the average relative abundance of *E. coli* in the healthy control group was 0.14 ± 0.5. Therefore, we define an *E. coli* colonization abundance less than 0.14 as “low-abundance colonization” and greater than 0.14 as “high-abundance colonization”. It was found that 40% of the healthy controls were high-abundance colonized populations, while the proportions for NAFLD and NASH patients were 80% and 77.8%, respectively (**Figure 1B**).

We selected and isolated *E. coli* strains from the colonic mucosa of the NASH patient who had the highest abundance of *Escherichia* (**Table 3** and **Figures S1B-E**). The genotypic identification of the above *E. coli* strains was carried out by full-length 16S rRNA sequencing of all 100 strains. The sequences showed 99% similarity with *E. coli* in the NCBI database. To analyze the evolutionary relationships among these strains, a phylogenetic tree was established using the maximum-likelihood method. The results showed that the 100 strains grouped into 3 clusters, which were named *E. coli* NF73-1 to NF73-73, *E. coli* NF23-1 to NF23-23 and *E. coli* NF4-1 to NF4-4 (**Figure S1F**). The 16S rRNA sequence of different *E. coli* NF strains have been uploaded to the NCBI GenBank database (MT649758-MT649857). Thus, *E. coli* NF73-1-NF73-73 were the dominant strains of *Escherichia* in the colonic mucosa of NASH patients. Therefore, *E. coli* NF73-1 was chosen for genome sequencing and verification of the pathogenic effect on NAFLD progression.

**Table 3:** Clinical characteristics of a NASH patient.

Age	52	Ultrasound examination: The liver was plump, smooth, with dull edge and slightly enhanced parenchyma echo. Imaging conclusion: Fatty liver
Sex	Female	
Weight, kg	73	
Height, m	1.61	
BMI, kg/m^2^	**28.16**	
Drinking	No	
ALT, IU/L	**129**
AST, IU/L	**75**	CT examination: The liver density was uniformly decreased, with a CT value of about 29.6 HU, which was lower than the intrahepatic vascular shadow. Imaging conclusion: Fatty liver
CHO, mmol/L	**7.21**	
TG, mmol/L	**4.26**	
Fasting glucose, mmol/L	6.03	
	
RBC, 10^12^/L	4.84	
WBC, 10^9^/L	9.56	
Platelets, 10^9^/L	248	
	
Hypertension	Yes	
Diabetes	No	
Tumor	No	
Virus hepatitis	No	

Bold values mean higher than normal standard.

### Whole-Genome Sequencing Analysis of the *E. coli* NF73-1 Strain

The genome of *E. coli* NF73-1 (authorized patent in China: No. 201610591293.4) was sequenced using an Illumina HiSeq 4000 system (Illumina, San Diego, CA, USA) at the Beijing Genomics Institute (Shenzhen, China). All experiments were conducted by HUADA GENE Company (Project Code. F17FTSNCKF1064_ECOaxmR). The sequenced reads were assembled using SOAPdenovo v1.05 software, leading to a final assembly of 113 contigs with an N50 of 75,968 bp (**Table 4**). The results of genomic component information analysis (Seq type scaffold) are presented in **Table 5**. The values for total length values were 4,626,248 (scaffold) and 4,612,049 (contig) depending on the seq type. The genomic sequence of *E. coli* NF73-1 has been uploaded to the SRA database (BioProject ID: PRJNA529543).

**Table 4:** Genomic assembly information of *E.coli* NF73-1.

Sample name	Seq type	Total number	Total length (bp)	N50 length (bp)	N90 length (bp)	Max length (bp)	Min length (bp)	Gap number	GC component (%)
*E. coli* NF73-1	Scaffold	108	4,626,248	75,968	25,983	261,941	517	1,135,730	50.73
	Contig	113	4,612,049	75,968	23,662	261,941	381	–	50.73

**Table 5:** General genome information of *E.coli* NF73-1.

Sample name	Genome size (Seq Type: Scaffold)	Total number	Total length (bp)	Average length	Total length/Genome size (%)	GC content (%)
*E. coli* NF73-1	4,626,248	4,372	4,036,362	923.23	87.25	51.85

The phylogenetic tree constructed by TreeBeST showed that *E. coli* NF73-1 was distinguished from other strains and shared relatively close evolutionary relationships with *E. coli* ETEC.H10407 and *E. coli* K12.MG1655 (**Figure 1C**). These data suggested that *E. coli* NF73-1, which was isolated from the intestinal mucosa of NASH patients, possessed several critical and pathogenic genes and differed from known strains. The analysis is shown in the **Table S1**. The genomes were assembled and annotated, and cluster analysis was performed (**Figure 1D**). A total of 4372 genes were obtained, of which 3241 were successfully annotated in KEGG databases (**Figure S2A** and **Table S2**). Through comparative genomics analysis with *E. coli* MG1655, it was found that virulence genes related to adhesion and invasion were more abundant in *E. coli* NF73-1 than *E. coli* MG1655. Virulence genes related to secretion system, manganese/iron transport system etc. only existed in *E. coli* NF73-1 (**Figure 1E** and **Table S3**).

In addition, the abundance of *Escherichia* was commonly increased in not only the stool samples but also the intestinal mucosa of NASH patients (Jiang et al., 2015). We speculated that the highly abundant *E. coli* NF73-1 may be an important pathogen causing NAFLD progression.

### *E. coli* NF73-1 Aggravated Hepatosteatosis and Inflammatory Cell Infiltration in NAFLD Mice

**Figure 2:**
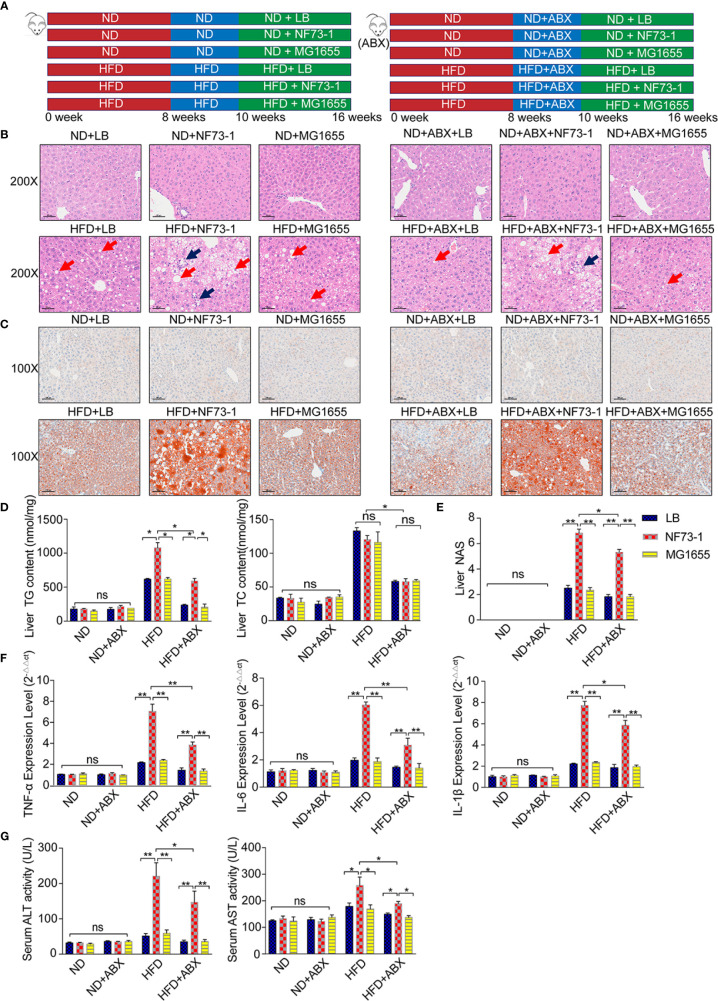
Liver injury enhancement in NAFLD mice with *E. coli* NF73-1. **(A)** Schematic diagram showing the overall design and complete timeline for model construction. **(B)** Representative images of H&E-stained mouse liver (blue arrows, inflammatory cells; red arrows, steatosis); scale bar indicates 50 μm. **(C)** Oil Red O-stained liver images; scale bar indicates 100 μm. **(D)** Hepatic triglyceride and cholesterol content and their concentrations in serum in each group. **(E)** NAS analysis of the liver. **(F)** TNF-α, IL-6, and IL-1β content in the liver in each group. **(G)** Serum ALT and AST activity in each group. Data are calculated as the mean ± SEM, n = 6–8 per group. ns p>0.05, *p <0.05, **p <0.01 (unpaired t test). NAFLD, non-alcoholic fatty liver disease; ABX, broad-spectrum antibiotics complex; ALT, alanine aminotransferase; AST, aspartate aminotransferase; TC, total cholesterol; TG, total triglyceride; H&E, hematoxylin & eosin; NAS, NAFLD activity score; TNF-α, tumor necrosis factor α; IL-6, interleukin 6; IL-1β, interleukin 1β; ND, normal diet; HFD, high-fat diet; NS, no significance.

Considering the enrichment of *E. coli* NF73-1 in the intestinal mucosa sample, we investigated the potential effect of this particular bacterial strain on NAFLD progression (**Figure 2A**). To further confirm whether *E. coli* NF73-1 independently caused the progression of NAFLD, we utilized flora-deficient mice in our gavage model to exclude the potential impact of other symbiotic gut microbes and repeat the experiments. None of the groups showed abnormal signs or symptoms. *E. coli* NF73-1 did not result in mouse death. *E. coli* NF73-1-treated HFD (**Table 6**) mice showed significantly higher weight gain than nontreated mice (**Figure S2B**) We did not observe significant differences in weight gain in ND mice harboring high *E. coli* NF73-1 abundance.

**Table 6:** Components of high fat diet.

Ingredient	MD12032 (g%/kcal%)
Protein	24/20
Fat	24/45
Carbohydrate	41/35
Microelement	11/0

Both small and large lipid droplet vacuoles within hepatocytes were significantly increased in the HFD-NF73-1 group compared with the HFD-LB and HFD-MG1655 groups, which was confirmed by liver H&E and Oil Red O staining (**Figures 2B, C**). Notably, only the HFD-NF73-1 group exhibited much higher hepatic triglyceride levels than paired-fed HFD mice (**Figure 2D**).

Liver H&E staining showed that *E. coli* NF73-1 administration resulted in inflammatory cell infiltration in the HFD-NF73-1 group compared with paired-fed HFD mice (**Figure 2B**). The HFD-NF73-1 group showed a higher NAFLD activity score (NAS) than the HFD-LB and HFD-MG1655 groups (**Figure 2E**). Moreover, real-time PCR analysis showed that the HFD-NF73-1 group had higher expression of inflammatory cytokines, including interleukin 6 (IL-6), interleukin 1β (IL-1β), and tumor necrosis factor α (TNF-α), in the liver than the HFD-LB and HFD-MG1655 groups (**Figure 2F**). Importantly, significant increases in ALT and AST were observed in the HFD-NF73-1 group compared with the HFD-LB and HFD-MG1655 groups (**Figure 2G**). No liver steatosis or inflammation was found among the ND-LB, ND-NF73-1, ND-MG1655, ND-ABX-LB, ND-ABX-NF73-1, and ND-ABX-MG1655 groups (**Figures 2B–F**).

After antibiotic cocktail treatment, bacterial abundance decreased significantly in stool and was maintained at a very low level (**Figure S3A**), indicating the establishment of natural gut flora depletion. Consistent with the conventional mouse model, elevated hepatic inflammation levels, triglyceride content and serum liver enzymes were also observed in the HFD-ABX-NF73-1 group compared with the paired-fed HFD-ABX mice (**Figures 2B–G**).

### Translocation of *E. coli* NF73-1 via the Gut-Liver Axis Is Observed in HFD but Not ND Mice

**Figure 3:**
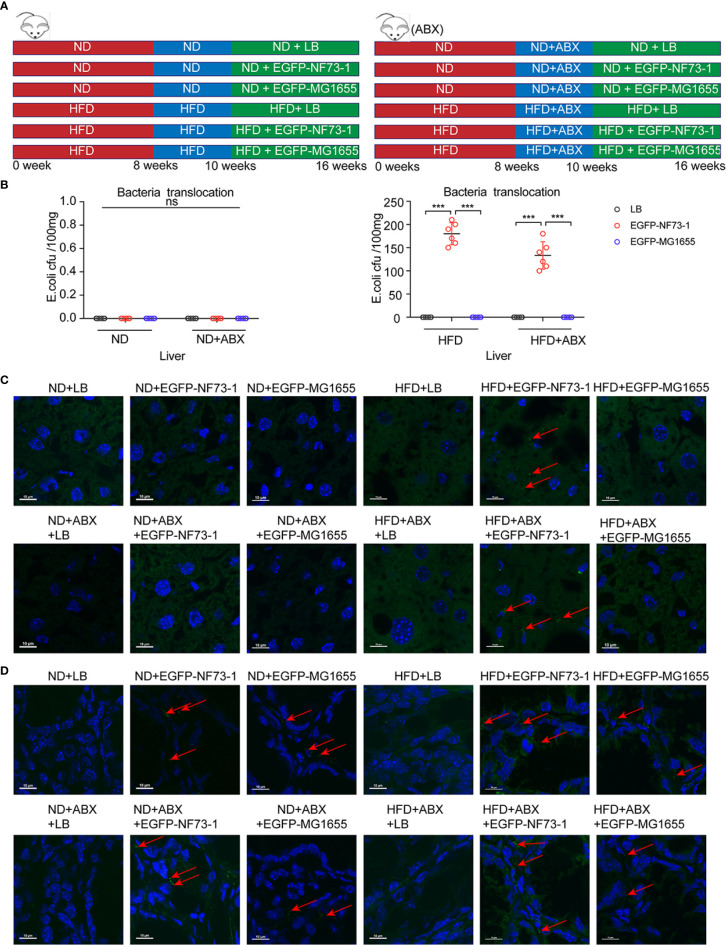
Translocation of *E. coli* EGFP-NF73-1 into the liver in the HFD-NF73-1 and HFD-ABX-NF73-1 groups and not in the ND-NF73-1 and ND-ABX-NF73-1 groups. **(A)** Schematic illustration of treatment with *E. coli* EGFP-NF73-1, *E. coli* EGFP-MG1655 and LB in ND and HFD mice. **(B)** Bacterial counts at specific points after oral gavage of NAFLD mice with 10^8^ *E. coli* EGFP-NF73-1 CFU per 0.1 g of liver. **(C)** Liver section assessment for *E. coli* EGFP-NF73-1 in mouse experiments. Highly notable EGFP signal in the livers of the HFD-NF73-1 and HFD-ABX-NF73-1 groups (red arrows, EGFP signal). The white scale bar indicates 10 μm. **(D)** Intestinal section assessment for *E. coli* EGFP-NF73-1 in mouse experiments (red arrows, EGFP signal); the white scale bar indicates 10 μm. Data are calculated as the mean ± SEM, n = 6 per group. ns p>0.05, ***p <0.001(unpaired t test). EGFP, enhanced green fluorescent protein; HFD, high-fat diet; ND, normal diet; NAFLD, non-alcoholic fatty liver disease. NS, no significance.

To follow bacterial translocation, EGFP-labeled, ampicillin-resistant *E. coli* EGFP-NF73-1 and *E. coli* EGFP-MG1655 were generated and used in animal experiments (**Figure S3B** and **Figure 3A**).

Notably, in the ND-EGFP-NF73-1, ND-ABX-EGFP-NF73-1, ND-EGFP-MG1655, and ND-ABX-EGFP-MG1655 groups, *E. coli* EGFP-NF73-1 and *E. coli* MG1655 were detected in the intestine but not in the liver, as confirmed by immunofluorescence and bacterial culture experiments (**Figures 3B–D**). However, in the HFD-EGFP-NF73-1 and HFD-ABX-EGFP-NF73-1 groups, *E. coli* EGFP-NF73-1 was detected not only in the intestine but also in the liver (**Figures 3B–D**).

Therefore, regardless of antibiotic cocktail treatment*, E. coli* EGFP-NF73-1 can adhere to the intestinal mucosa and translocate into the liver in NAFLD mice, but not in ND mice, via the gut-liver axis.

### *E. coli* NF73-1 Promoted M1 Macrophage Polarization in NAFLD Mice

We subsequently attempted to identify the liver immune cell subtypes of NAFLD mice by flow cytometry analysis after *E. coli* NF73-1 administration.

**Figure 4:**
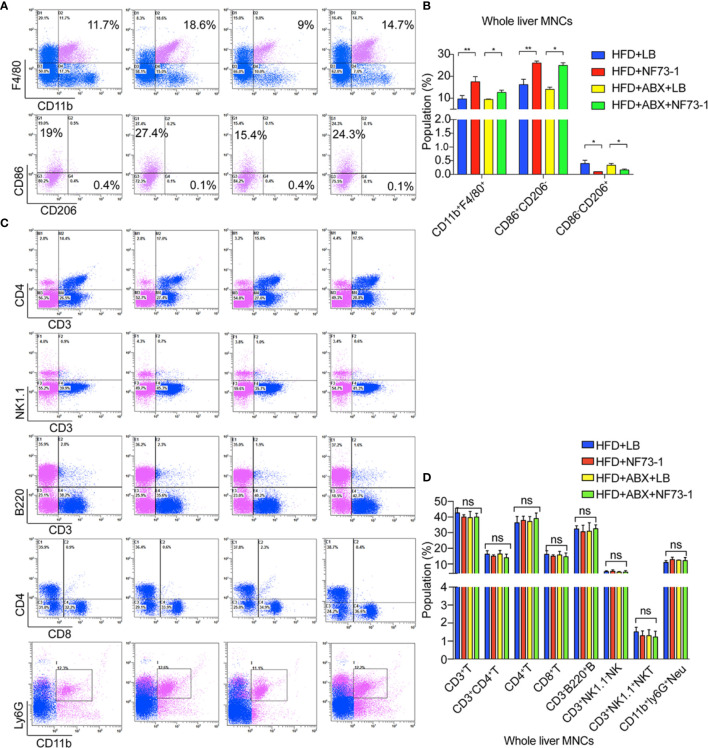
M1 macrophage polarization in NAFLD mice with *E. coli* EGFP-NF73-1. **(A)** Representative flow cytometric plots of the CD45^+^F4/80^+^CD11b^+^ liver macrophage population, M1(CD45^+^F4/80^+^CD11b^+^CD206^-^CD86^+^) and M2(CD45^+^F4/80^+^CD11b^+^CD86^-^CD206^+^) in the livers of the HFD-NF73-1, HFD-LB, HFD-ABX-NF73-1, and HFD-ABX-LB groups; **(B)** Liver macrophage counts in the livers of the HFD-NF73-1, HFD-LB, HFD-ABX-NF73-1, and HFD-ABX-LB groups; **(C)** Representative flow cytometric plots of the liver lymphocyte and neutrophil populations in the livers of the HFD-NF73-1, HFD-LB, HFD-ABX-NF73-1, and HFD-ABX-LB groups; **(D)** Lymphocyte and neutrophil counts in the livers of the HFD-NF73-1, HFD-LB, HFD-ABX-NF73-1, and HFD-ABX-LB groups. Data are calculated as the mean ± SEM, n = 4–6 per group. ns p>0.05, *p <0.05, **p <0.01 (unpaired t test). NAFLD, non-alcoholic fatty liver disease; HFD, high-fat diet.; NS, no significance.

Notably, the population of CD45^+^CD11b^+^F4/80^+^ macrophages was significantly increased in the HFD-NF73-1 group compared with the HFD-LB group (**Figures 4A, B**). Flow cytometry analyses revealed no changes in hepatic lymphocyte and neutrophil populations between the HFD-NF73-1 and HFD-LB groups (**Figures 4C, D**). Furthermore, *E. coli* NF73-1 led to a sharp increase in M1 (CD45^+^ly6G^-^F4/80^+^CD11b^+^CD86^+^CD206^-^) macrophages and a decrease in M2 (CD45^+^ly6G^-^F4/80^+^CD11b^+^CD206^+^CD86^−^) macrophages, indicating preponderant M1 polarization in the HFD-NF73-1 group (**Figures 4A, B**). In addition, these effects were also observed in flora-deficient HFD mice (**Figures 4A–D**). Collectively, *E. coli* NF73-1 might promote the progression of steatohepatitis by acting on hepatic M1 macrophage polarization.

### Depletion of Macrophages Attenuates *E. coli* NF73-1-Induced Hepatic Inflammation and Hepatosteatosis

**Figure 5:**
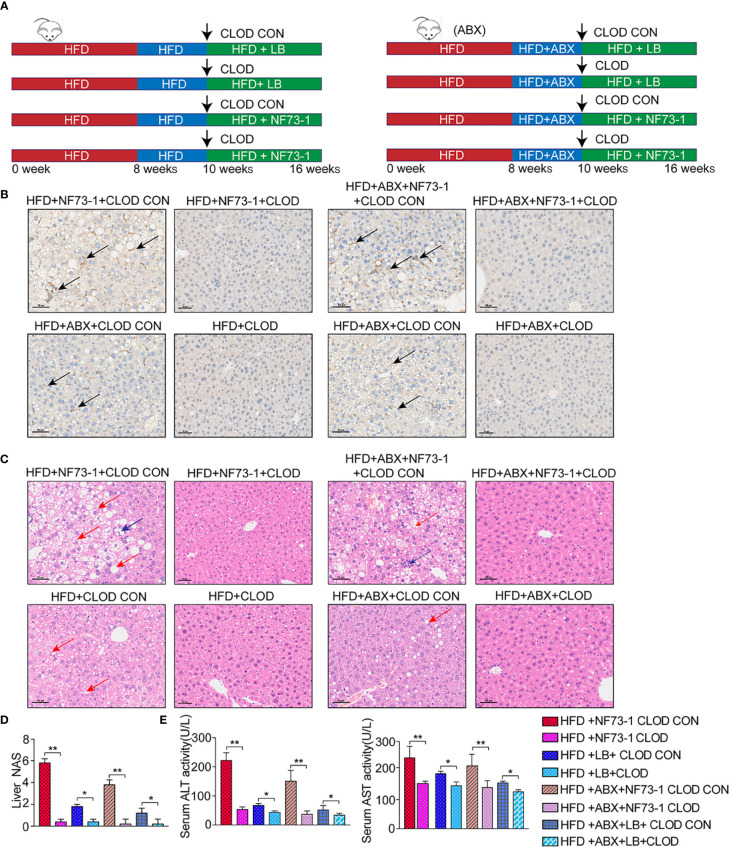
Pharmacological CLOD liposomes prevented *E. coli* NF73-1–induced steatohepatitis in NAFLD mice. **(A)** Schematic illustration of effective CLOD liposomes and control CLOD liposomes in the HFD and HFD-ABX groups with or without treatment with *E. coli* NF73-1. **(B)** Representative immunohistochemistry images for F4/80 in livers from the HFD-NF73-1, HFD-LB, HFD-ABX-NF73-1, and HFD-ABX-LB groups depleted of macrophages by CLOD treatment. **(C)** Representative H&E images of livers (blue arrows, inflammatory cells; red arrows, steatosis); black scale bar indicates 50 μm. **(D)** NAS analysis of liver samples. **(E)** Sreum ALT and AST in each group. Data are calculated as the mean ± SEM, n = 4–6 per group. *p <0.05, **p <0.01 (unpaired t test). NAFLD, non-alcoholic fatty liver disease; HFD, high-fat diet; ALT, alanine aminotransferase; AST, aspartate aminotransferase; H&E, hematoxylin & eosin; NAS, NAFLD activity score; CLOD, clodronate liposome; CLOD CON, clodronate liposome control.

We treated HFD and flora-deficient HFD mice with liposome-encapsulated clodronate (CLOD) to deplete macrophages or liposome-encapsulated PBS as a negative control (**Figure 5A**). Macrophages were significantly depleted by injection of CLOD in the HFD-NF73-1 and HFD-ABX-NF73-1 groups (**Figure 5B**). Liver H&E staining revealed that the depletion of polarized macrophages could significantly reduce hepatic steatosis and inflammation (**Figure 5C**). Liver enzymes and NAS were dramatically alleviated in the HFD-NF73-1 and HFD-ABX-NF73-1 groups treated with CLOD (**Figures 5D, E**). These results suggested that removing polarized M1 macrophages significantly ameliorated the liver damage and lipid abnormalities caused by *E. coli* NF73-1 in NAFLD mice.

### *E. coli* NF73-1 Induces RAW264.7 Macrophage M1 Polarization by Activating Toll-Like Receptor 2 (TLR2)-NF-κB/NLRP3-Caspase-1 Signaling *In Vitro*

**Figure 6:**
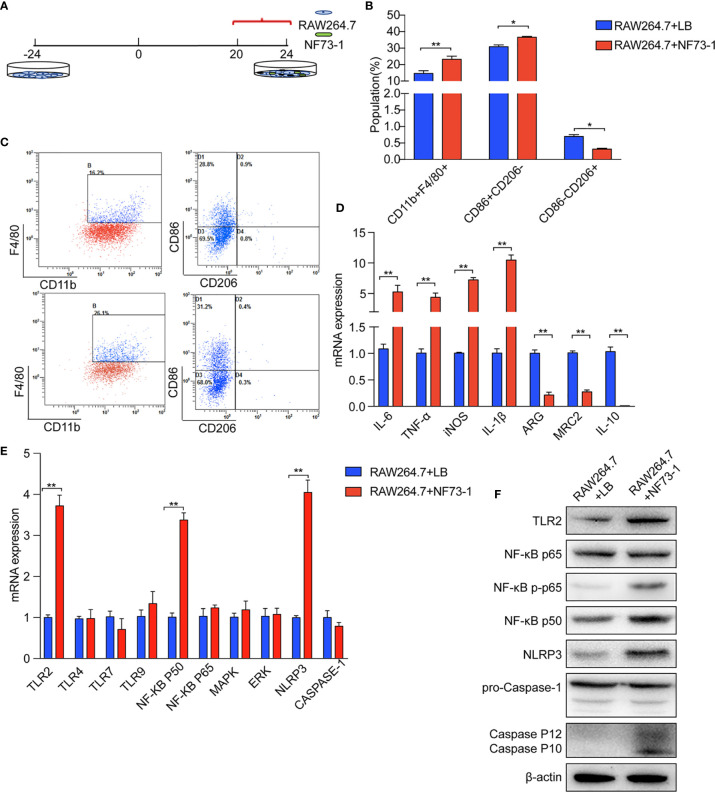
*E. coli* NF73-1, the inducer of M1 macrophage polarization. **(A)** RAW264.7 cells exposed to *E. coli* NF73-1 (multiplicity of infection = 1:10) for 4 h. **(B)** Effects of *E. coli* NF73-1 on the expression of CD11b, F4/80, CD86, and CD206 in RAW264.7 cells. RAW264.7 cells were cultured with palmitic acid and oleic acid for 20 h without *E. coli* NF73-1. **(C)** Representative flow plots. **(D)** cDNA expression of M1 and M2 markers after coculture with RAW264.7 and *E. coli* NF73-1 cells. **(E)** mRNA expression in RAW264.7 cells. **(F)** Protein synthesis in RAW264.7 cells. Data are calculated as the mean ± SEM, *p <0.05, **p <0.01 (unpaired t test). TNF-α, tumor necrosis factor α; IL-6, interleukin 6; IL-1β, interleukin 1β; iNOS, inducible nitric oxide synthase; Arg, arginase; Mrc2, macrophage mannose receptor 2, IL-10, interleukin 10; TLR, toll-like receptor; NF-KB, nuclear factor kappa-B; MAPK, mitogen-activated protein kinase; ERK, extracellular regulated protein kinases; NLRP3, Nod-like receptor pyrin domain containing 3.

We validated the effect of *E. coli* NF73-1 on M1 polarization *in vitro* (**Figure 6A**). We found that the proportion of M1 (CD11b^+^F4/80^+^CD86^+^CD206^-^) macrophages markedly increased after treatment with *E. coli* NF73-1 (**Figures 6B, C**). Moreover, after coculture with *E. coli* NF73-1, RAW264.7 macrophages showed increased expression of most proinflammatory cytokines (TNF-α, IL-6, IL-1β, and iNOS) and decreased expression of tissue repair-related M2 genes (Arg1, Mrc2, and IL-10) (**Figure 6D**). The cytokine secretion profiles suggested that *E. coli* NF73-1 functioned as a proinflammatory agent and induced M1 polarization.

We found that *E. coli* NF73-1 stimulation upregulated the mRNA expression of TLR2, nuclear factor-κB (NF-κB) p50, and Nod-like receptor pyrin domain containing 3 (NLRP3), as well as the protein level (**Figures 6E, F**). *E. coli* NF73-1 stimulation upregulated the phosphorylation of NF-κB P65 and Cleaved-Caspase-1 on RAW264.7 macrophages (**Figure 6F**). These results strongly suggested that *E. coli* NF73-1 directly promotes the activation and polarization of RAW264.7 macrophages via the TLR2-NF-κB/NLRP3-Caspase-1 pathway.

### *E. coli* NF73-1 Induces the Disruption of Triglyceride Synthesis and Oxidation in NAFLD Mice

**Figure 7:**
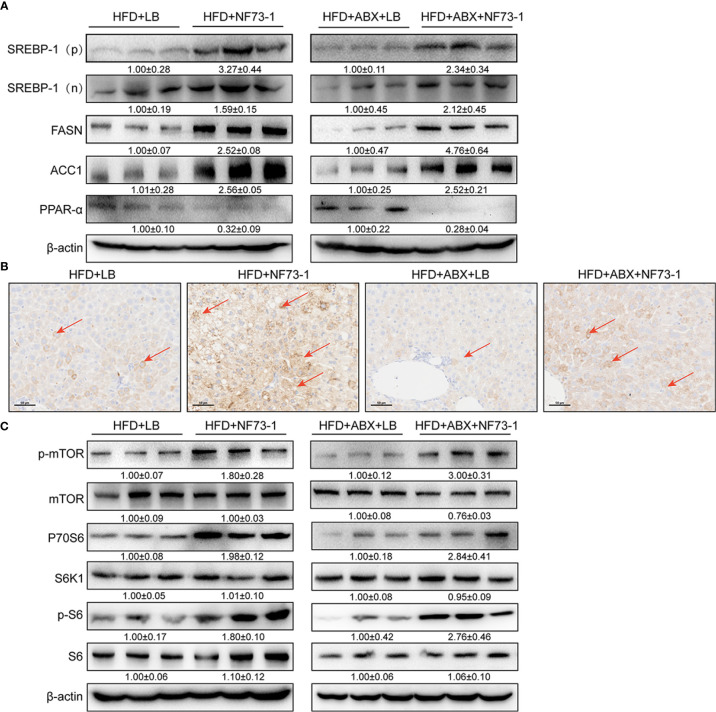
Activation of mTOR signaling and promotion of NAFLD in *E. coli* NF73-1–fed mice. **(A)** Increasing nuclear translocation of SREBP-1(N) and expression of SREBP-1 (P), as well as its targets, such as ACC1 and FASN, after *E. coli* NF73-1 treatment and decreasing PPAR-α expression after *E. coli* NF73-1 treatment. **(B)** Positive immunostaining for phosphorylated S6 (brown color), the predominant location in lipid-rich hepatocytes (red arrows) of NAFLD mice. **(C)** Representative immunoblots of phosphorylation of mTOR, S6K1, and S6 in livers from three mice in each group. Total liver lysates of the indicated genotypes were subjected to western blot analysis and densitometry analysis. Data are means ± SEM (n = 3). NAFLD, non-alcoholic fatty liver disease; mTOR, mammalian target of rapamycin; N, cleaved nuclear (68 kDa) form of SREBP-1; P, precursor (125 kDa) form of SREBP-1; S6K1, S6 kinase 1; SREBP-1, sterol-regulatory element binding protein-1; ACC1, acetyl-coenzyme A carboxylase; FASN, fatty acid synthase; PPAR-α, peroxisome proliferator-activated receptor alpha.

An excess of fat accumulation was also accompanied by upregulation of sterol regulatory element binding protein-1 (SREBP-1) and downregulation of peroxisome proliferator-activated receptor alpha (PPAR-α) (Chen et al., 2018). *E. coli* NF73-1-mediated upregulation of SREBP-1 and its target genes, including acetyl-coenzyme A carboxylase (ACC1) and fatty acid synthase (FAS), was significantly higher than in the HFD-LB group (**Figure 7A**). Moreover, Western blot analysis showed that *E. coli* NF73-1 exposure caused significant impairment in fatty acid oxidation, as evidenced by decreased expression of the nuclear receptor PPAR-α (**Figure 7A**).

Furthermore, immunohistochemical analysis revealed that elevated positive signals of S6 phosphorylation were colocalized primarily in the cytoplasm of lipid droplet-rich hepatocytes of the HFD-NF73-1 group (**Figure 7B**). Constitutively activated mTOR greatly elevates de novo lipid synthesis (Duvel et al., 2010; Chen et al., 2018). Phosphorylation of mammalian target of rapamycin (mTOR) at Ser2448 was increased in the HFD-NF73-1 group compared with the HFD-LB group (**Figure 7C**). Thr389 phosphorylation of S6K1, which is modulated by mTOR, was increased. The phosphorylation of the S40 ribosomal protein S6 at Ser235/Ser236, a well-characterized substrate of S6K1 (S6 kinase 1), was increased (**Figure 7C**).

In addition, regardless of whether antibiotics were used, the expression of the above genes presented a consistent trend. Collectively, *E. coli* NF73-1-mediated disruption of mTOR-S6K1-SREBP-1/PPAR-α signaling caused the disruption of triglyceride synthesis and oxidation in NAFLD mice.

### M1 Macrophages Stimulated by *E. coli* NF73-1 Enhance Triglyceride Accumulation via the mTOR/S6K1-Dependent Pathway in Hepatocytes

We investigated whether *E. coli* NF73-1 is involved in regulating triglyceride accumulation in hepatocytes following translocation (**Figure S4A**). We proposed that *E. coli* NF73-1 is a promoting factor of triglyceride accumulation in hepatocytes. However, lipid accumulation in oleic acid- and palmitic acid-induced L02 and H2.35 cells treated with or without *E. coli* NF73-1 was similar (**Figure S4B**). *E. coli* NF73-1 cannot restore the expression of lipolytic genes (PPAR-α, SREBP-1 and p-mTOR) in hepatocytes (**Figure S4C**). Our results suggest that *E. coli* NF73-1 could not directly promote lipid accumulation in hepatocytes.

**Figure 8:**
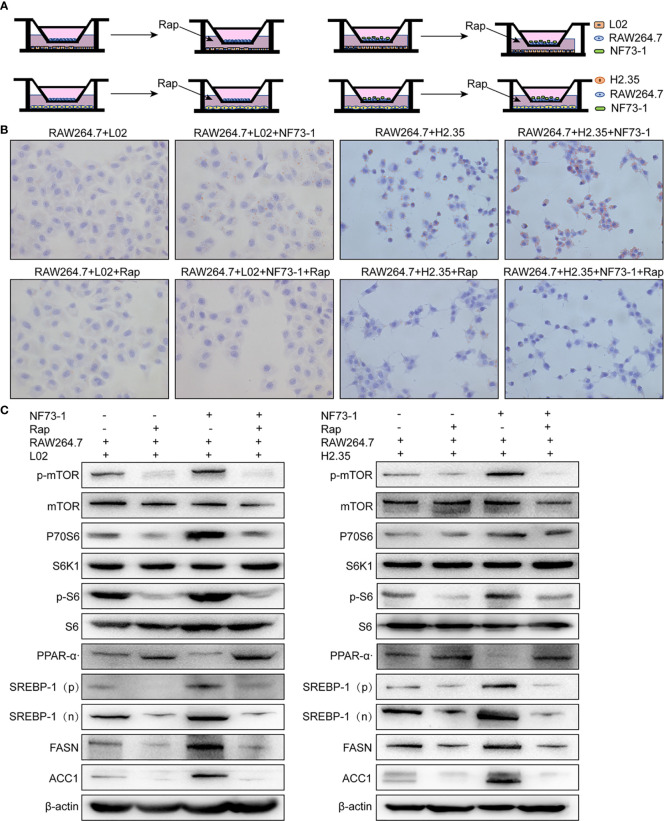
*E. coli* NF73-1, the inducer of M1 polarization and hepatocyte steatohepatitis *in vitro.* **(A)** Schematic illustration of L02 and H2.35 hepatocytes with RAW264.7, in the presence or absence *E. coli* NF73-1 and rapamycin with palmitic acid- and oleic acid-containing medium. **(B)** Lipid accumulation detected by Oil Red O staining in hepatocytes cocultured with RAW264.7 in the presence or absence *E. coli* NF73-1 and rapamycin with palmitic acid- and oleic acid-containing medium. **(C)** Protein synthesis of phosphorylated mTOR, S6K1, S6, SREBP-1, ACC1, FASN, and PPAR-α in palmitic acid- and oleic acid-treated hepatocytes cocultured with or without *E. coli* NF73-1 and rapamycin. mTOR, mammalian target of rapamycin; S6K1, S6 kinase 1; SREBP-1, sterol-regulatory element binding protein-1; ACC1, acetyl-coenzyme A carboxylase; FASN, fatty acid synthase; PPAR-α, peroxisome proliferator-activated receptor alpha; N, cleaved nuclear (68 kDa) form of SREBP-1; P, precursor (125 kDa) form of SREBP-1.

*In vitro*, *E. coli* NF73-1 enriched lipid accumulation in oleic acid- and palmitic acid-induced L02 and H2.35 cells via M1 polarized RAW264.7 macrophages (**Figure 8A**), respectively, which was more significant than the accumulation observed in the non-*E. coli* NF73-1 coculture group. The expression of p-mTOR and its downstream genes was altered consistently between the 2 cell lines, leading to increased lipid synthesis and reduced lipid oxidation catabolism (**Figures 8B, C**). These pathways coincide with those observed in NAFLD mice, consistent with lipid dysmetabolism. After adding rapamycin, an inhibitor of p-mTOR, the lipid accumulation and gene expression profiles were significantly reversed and showed consistent changes in the two cell lines (**Figures 8B, C**).

These experiments showed that *E. coli* NF73-1 could increase triglyceride synthesis and reduce the catabolism of hepatocytes via the mTOR pathway through the activation of M1 macrophages.

## Discussion

The gut microbiota has emerged as a key player in NAFLD (Le Roy et al., 2013; Safari and Gerard, 2019). Increasing evidence has demonstrated a difference in gut microbiota composition between NAFLD patients and healthy controls (Ley et al., 2006; Jiang et al., 2015; Schwimmer et al., 2019). However, previous studies have seldom focused on one specific bacterial strain in the investigation of NAFLD pathogenesis. Therefore, little data available from animal models showing that changes in gut bacterial strains contribute to NAFLD. More importantly, deeper insight into how the gut microbiota influences NAFLD at the strain level is still lacking.

Different strains of different bacterial species have different effects and clinical manifestations in human hosts with NAFLD. *Klebsiella pneumoniae*, a high-alcohol-producing strain, causes autobrewery syndrome (ABS) and hepatic mitochondrial dysfunction (Yuan et al., 2019). *Enterobacter cloacae* B29, an opportunistic pathogen, causes obesity in mice via insulin resistance (Fei and Zhao, 2013; Yan et al., 2016). Even different strains of the same bacterial species have different effects in the human host. As shown in the present study, liver injury was not induced by a nonpathogenic control bacterial strain (*E. coli* MG1655). Indeed, the mechanisms in NAFLD development, which are launched by specific strains of gut microbiota, need to be further explored.

In this study, we also found that the *Escherichia* abundance increased in NAFLD patients, not only in feces but also in the gut mucosa. *E. coli* NF73-1, the most highly enriched strain of *Escherichia* in NAFLD and NASH patients, was successfully isolated from the intestinal mucosa of NASH patients, and its effect on NAFLD progression was confirmed in animal experiments. Several liver diseases have been shown to be influenced by PBT from the gut (Fouts et al., 2012; Manfredo Vieira et al., 2018), such as autoimmune liver disease (Manfredo Vieira et al., 2018), cholestatic liver injury (Liu et al., 2018; Liao et al., 2019), liver cirrhosis (Sorribas et al., 2019) and NAFLD (Wu et al., 2018). We revealed that *E. coli* NF73-1 translocated into the liver of bacteria-depleted NAFLD mice, which also supported the conclusion regarding PBT. Interestingly, *E. coli* NF73-1 translocated only into the liver of NAFLD mice. We inferred that this was based on damage to the intestinal mechanical barrier of NAFLD mice (Peterson and Artis, 2014), as well as the immune barrier (Bibbo et al., 2018), intestinal vascular barrier (Mouries et al., 2019) and even liver barrier (Balmer et al., 2014).

We further confirmed that the liver immune response, which was activated by *E. coli* NF73-1 exposure, was related to the progression from NAFLD to NASH. The data showed that liver macrophages played a critical role in the pathogenic mechanisms of NASH induced by *E. coli* NF73-1. Liver macrophages exhibit heterogeneity and plasticity in the tissue microenvironment, especially under pathological conditions such as steatohepatitis (Sica et al., 2014). Macrophage infiltration is an early event in the development of NASH and is associated with progressive disease (Gadd et al., 2014). Microbial detection requires the recognition of pathogen-associated molecular patterns (PAMPs) by pattern recognition receptors (PRRs) in innate immune cells (Himes and Smith, 2010; Csak et al., 2011; Jenne and Kubes, 2013; Miura et al., 2013; Mridha et al., 2017). Previous studies have found that macrophages activate local inflammatory responses and cytokine production, and the coactivation of TLR2 and free fatty acids leads to the progression of NASH (Spruss et al., 2009; Miura et al., 2013; Roh and Seki, 2013). Additionally, the NLRP3 inflammasome has been reported to be involved in bacterial recognition as well as macrophage activation. Complex lipids of bacterial origin trigger inflammation through the binding of NLRP3 to induce Caspase-1 cleavage in macrophages (Vandanmagsar et al., 2011). Taken together, the results indicated that *E. coli* NF73-1 induced the activation of the TLR2-NF-KB/NLRP3-Caspase-1 signaling pathway to mediate the polarization of macrophages.

The different polarization states of macrophages are correlated with their functional diversity (Chawla et al., 2011). Macrophages are generally classified as M1 and M2 phenotypes. Evidence from both human and animal studies suggests that during the progression of NASH, excess metabolites and bacterial products induce macrophage M1 polarization (Itoh et al., 2013; Lotowska et al., 2013; Gadd et al., 2014). In our study, we demonstrated that the M1 polarization induced by *E. coli* NF73-1 was responsible for the progression of NAFLD. The above findings from animal experiments were further confirmed by *in vitro* assays. Hence, our data suggested that *E. coli* NF73-1 was involved in triggering of inflammation in macrophages. Abnormal metabolism of triglycerides is also an important factor in the pathogenesis of NAFLD (Kawano and Cohen, 2013). Previous studies have reported that the mTOR signaling pathway regulates lipid metabolism (Ai et al., 2012). With obesity, overnutrition and alcoholic liver disease (ALD), mTOR is hyperactivated, resulting in persistent activation of SREBP-1 in the liver (Ai et al., 2012). Based on our data, triglyceride metabolism disruption, which was induced by *E. coli* NF73-1, tended to be an important factor in NAFLD development. We discovered that the mTOR pathway was triggered by *E. coli* NF73-1 in mice with NAFLD, which is characterized by an increase in mTOR-mediated phosphorylation and activity of S6K1 and S6. We speculate that activation of the mTOR signaling pathway by *E. coli* NF73-1 might be the mechanism of NAFLD aggravation. Collectively, our data suggested that *E. coli* NF73-1 switched macrophage polarization to the M1 phenotype, inducing hepatocyte lipolysis.

Numerous studies have shown that gut dysbiosis is a pathogenetic factor that plays a significant role in the development and progression of liver diseases. Different pathogenic bacteria have been found to exert distinct effects in different liver diseases. It is necessary to identify pathogenic bacteria and functional bacteria, which might be a novel target for the prevention and diagnosis of liver diseases. However, most previous studies focused on the disturbance of the gut flora at only the genus and species levels; they did not illustrate how these pathogenic bacteria aggravate liver diseases at the strain level. Thus, more breakthroughs at the strain level are needed to obtain deeper insight into the prevention of liver diseases associated with the gut flora. In summary, the exploration of bacterial strains is a key point for investigating the mechanisms of liver diseases.

In this study, the pathogenic strain was successfully isolated from clinical intestinal mucosa samples. We demonstrated that translocation of intestinal *E. coli* NF73-1 into the liver was responsible for the high hepatic M1 population, which further aggravated liver injury, leading to disturbance of the hepatic triglyceride metabolism and, eventually, NAFLD progression. In conclusion, *E. coli* NF73-1 might be a critical trigger in the progression of NAFLD to NASH, and future clinical trials are needed to clarify the role of *E. coli* NF73-1 in a larger NAFLD population.

## Data Availability Statement

The 16S rRNA sequence of different *E. coli* NF strains have been uploaded to the NCBI GenBank database (MT649758-MT649857).

## Ethics Statement 

The studies involving human participants were reviewed and approved by the Ethics Committee of Peking University People’s Hospital. The patients/participants provided their written informed consent to participate in this study. The animal study was reviewed and approved by the Ethics Committee of Peking University People’s Hospital.

## Author Contributions

Supervision, reviewing and editing, resources: YLL. Writing, Original draft preparation: YFZ. Funding acquisition: YLL. Project administration: YLL. Investigation: YFZ and WWJ. Methodology, software, validation: YFZ, JX, and YW. Data curation, formal analysis: NW, TYL, and YL. All authors contributed to the article and approved the submitted version.

## Funding

This work was financially supported by grant from National Key Research and Development Program of China (Grant Number 2017YFC0908903) and the National Natural Science Foundation of China (Grant Number 81873549) and the National Natural Science Foundation of China (Grant Number 82070539).

## Conflict of Interest

The authors declare that the research was conducted in the absence of any commercial or financial relationships that could be construed as a potential conflict of interest.

## Supplementary Material

Supplementary Figure 1 Isolation and whole-genome sequencing of clinical *Escherichia* strains from the intestinal mucosa of NASH patients. **(A)** Comparison of the seven genera abundance among NASH and NAFLD patients and healthy subjects. **(B)** Schematic diagram of the overall design and bacterial isolation**. (C)** Two representative liver H&E images of NASH patients (blue arrows, inflammatory cells; red arrows, steatosis). **(D)** Negative immunostaining for HBsAg and HBcAg in the NASH patient. **(E)** Representative image of *Escherichia* strains on *E. coli* Chromogenic Medium. **(F)** Phylogenetic relationships among clinical *E. coli* strains inferred with the maximum likelihood method based on the full-length 16S rRNA sequence. The black scale bar indicates 100 μm and 50 μm. Data are calculated as the mean ± SEM, n = 9–10 per group. *p <0.05, **p <0.01 (one-way analysis of variance between multiple groups).

Supplementary Figure 2 General functional annotations of *E. coli* NF73-1 and body weight of mouse. **(A)** KEGG general functional annotations of *E. coli* NF73-1. **(B)** Body weight of mouse. Data are calculated as the mean ± SEM, n = 5 per group. *p <0.05, **p <0.01 (one-way analysis of variance between multiple groups).

Supplementary Figure 3 Total bacteria in stool samples with administration of antibiotics in NAFLD mice and the generation of *E. coli* EGFP-NF73-1. **(A)** Total bacterial loads in stool samples of NAFLD mice and ND mice treated with antibiotics were determined from the DNA concentrations. Data are calculated as the mean ± SEM, n = 4–6 per group. **p <0.01 (one-way analysis of variance between multiple groups). **(B)** Representative image of EGFP expression of *E. coli* EGFP-NF73-1 and *E. coli* EGFP-MG 1655 strains and the positive control. **(C)** Flow cytometry gating strategies for liver macrophages. Representative flow cytometric plots. **(D)** Flow cytometry gating strategies for liver lymphocytes. Representative flow cytometric plots; **(E)** Flow cytometry gating strategies for RAW264.7 macrophages. Representative flow cytometric plots.

Supplementary Figure 4
*E. coli* NF73-1, the non-inducer of lipid accumulation in hepatocytes *in vitro*. **(A)** The human liver normal cell line L02 and mouse liver normal cell line H2.35 were exposed to *E. coli* NF73-1 (multiplicity of infection = 1:10) for 4 h. **(B)** Lipid accumulation determined by Oil Red O staining of palmitic acid- and oleic acid-treated hepatocytes cocultured with or without *E. coli* NF73-1. **(C)** Protein synthesis of phosphorylated mTOR, S6K1, S6, SREBP-1, ACC1, FASN, and PPAR-α in palmitic acid- and oleic acid-treated hepatocytes cocultured with or without *E. coli* NF73-1. mTOR, mammalian target of rapamycin; S6K1, S6 kinase 1; SREBP-1, sterol-regulatory element binding proteins-1; ACC1, acetyl-coenzyme A carboxylase; FASN, fatty acid synthase; PPAR-a, peroxisome proliferator-activated receptor alpha; N, cleaved nuclear (68 kDa) form of SREBP-1; P, precursor (125 kDa) form of SREBP-1.

## Abbreviations

NAFLD, non-alcoholic fatty liver disease; ABX, broad-spectrum antibiotics complex; ALT, alanine aminotransferase; AST, aspartate aminotransferase; TC, total cholesterol; TG, total triglyceride; H&E, hematoxylin & eosin; NAS, NAFLD activity score; TNF-α, tumor necrosis factor α; IL-6, interleukin 6; IL-1β, interleukin 1β; ND, normal diet; HFD, high-fat diet; EGFP, enhanced green fluorescent protein; H&E, hematoxylin & eosin; iNOS, inducible nitric oxide synthase; Arg, arginase; Mrc2, macrophage mannose receptor 2, IL-10, interleukin 10; TLR, toll-like receptor; NF-KB, nuclear factor kappa-B; MAPK, mitogen-activated protein kinase; ERK, extracellular regulated protein kinases; NLRP3, Nod-like receptor pyrin domain containing 3; mTOR, mammalian target of rapamycin; N, cleaved nuclear form of SREBP-1; P, precursor form of SREBP-1; S6K1, S6 kinase 1; SREBP-1, sterol-regulatory element binding protein-1; ACC1, acetyl-coenzyme A carboxylase; FASN, fatty acid synthase; PPAR-α, peroxisome proliferator-activated receptor alpha; ABX, broad-spectrum antibiotics complex.

## References

[B1] Ai D. Baez J. M. Jiang H. Conlon D. M. Hernandez-Ono A. Frank-Kamenetsky M. (2012). Activation of ER stress and mTORC1 suppresses hepatic sortilin-1 levels in obese mice. J. Clin. Invest. 122, 1677–1687. 10.1172/JCI61248 22466652 PMC3336989

[B2] Backhed F. Ding H. Wang T. Hooper L. V. Koh G. Y. Nagy A. (2004). The gut microbiota as an environmental factor that regulates fat storage. Proc. Natl. Acad. Sci. U.S.A. 101, 15718–15723. 10.1073/pnas.0407076101 15505215 PMC524219

[B3] Balmer M. L. Slack E. de Gottardi A. Lawson M. A. E. Hapfelmeier S. Miele L. (2014). The Liver May Act as a Firewall Mediating Mutualism Between the Host and Its Gut Commensal Microbiota. Sci. Transl. Med. 6 (237), 237ra66. 10.1126/scitranslmed.3008618 24848256

[B4] Bibbo S. Ianiro G. Dore M. P. Simonelli C. Newton E. E. Cammarota G. (2018). Gut Microbiota as a Driver of Inflammation in Nonalcoholic Fatty Liver Disease. Mediators Inflammation 2018, 9321643. 10.1155/2018/9321643 PMC5833468 29563854

[B5] Boursier J. Mueller O. Barret M. Machado M. Fizanne L. Araujo-Perez F. (2016). The severity of nonalcoholic fatty liver disease is associated with gut dysbiosis and shift in the metabolic function of the gut microbiota. Hepatology 63, 764–775. 10.1002/hep.28356 26600078 PMC4975935

[B6] Brandt A. Jin C. J. Nolte K. Sellmann C. Engstler A. J. Bergheim I. (2017). Short-Term Intake of a Fructose-, Fat- and Cholesterol-Rich Diet Causes Hepatic Steatosis in Mice: Effect of Antibiotic Treatment. Nutrients 9(9), 1013. 10.3390/nu9091013 PMC5622773 28906444

[B7] Caporaso J. G. Kuczynski J. Stombaugh J. Bittinger K. Bushman F. D. Costello E. K. (2010). QIIME allows analysis of high-throughput community sequencing data. Nat. Methods 7, 335–336. 10.1038/nmeth.f.303 20383131 PMC3156573

[B8] Chalasani N. Younossi Z. Lavine J. E. Diehl A. M. Brunt E. M. Cusi K. (2012). The diagnosis and management of non-alcoholic fatty liver disease: practice guideline by the American Gastroenterological Association, American Association for the Study of Liver Diseases, and American College of Gastroenterology. Gastroenterology 142, 1592–1609. 10.1053/j.gastro.2012.04.001 22656328

[B9] Chawla A. Nguyen K. D. Goh Y. P. (2011). Macrophage-mediated inflammation in metabolic disease. Nat. Rev. Immunol. 11, 738–749. 10.1038/nri3071 21984069 PMC3383854

[B10] Chen S. L. Hung C. S. Pinkner J. S. Walker J. N. Cusumano C. K. Li Z. (2009). Positive selection identifies an in vivo role for FimH during urinary tract infection in addition to mannose binding. Proc. Natl. Acad. Sci. U.S.A. 106, 22439–22444. 10.1073/pnas.0902179106 20018753 PMC2794649

[B11] Chen H. Shen F. Sherban A. Nocon A. Li Y. Wang H. (2018). DEP domain-containing mTOR-interacting protein suppresses lipogenesis and ameliorates hepatic steatosis and acute-on-chronic liver injury in alcoholic liver disease. Hepatology 68, 496–514. 10.1002/hep.29849 29457836 PMC6097912

[B12] Chiu C. C. Ching Y. H. Li Y. P. Liu J. Y. Huang Y. T. Huang Y. W. (2017). Nonalcoholic Fatty Liver Disease Is Exacerbated in High-Fat Diet-Fed Gnotobiotic Mice by Colonization with the Gut Microbiota from Patients with Nonalcoholic Steatohepatitis. Nutrients 9 (11), 1220. 10.3390/nu9111220 PMC5707692 29113135

[B13] Chu H. Duan Y. Yang L. Schnabl B. (2019). Small metabolites, possible big changes: a microbiota-centered view of non-alcoholic fatty liver disease. Gut 68, 359–370. 10.1136/gutjnl-2018-316307 30171065

[B14] Csak T. Velayudham A. Hritz I. Petrasek J. Levin I. Lippai D. (2011). Deficiency in myeloid differentiation factor-2 and toll-like receptor 4 expression attenuates nonalcoholic steatohepatitis and fibrosis in mice. Am. J. Physiol. Gastrointest. Liver Physiol. 300, G433–G441. 10.1152/ajpgi.00163.2009 21233280 PMC3302188

[B15] Del Chierico F. Nobili V. Vernocchi P. Russo A. De Stefanis C. Gnani D. (2017). Gut microbiota profiling of pediatric nonalcoholic fatty liver disease and obese patients unveiled by an integrated meta-omics-based approach. Hepatology 65, 451–464. 10.1002/hep.28572 27028797

[B16] Duvel K. Yecies J. L. Menon S. Raman P. Lipovsky A. I. Souza A. L. (2010). Activation of a metabolic gene regulatory network downstream of mTOR complex 1. Mol. Cell 39, 171–183. 10.1016/j.molcel.2010.06.022 20670887 PMC2946786

[B17] Edgar R. C. Haas B. J. Clemente J. C. Quince C. Knight R. (2011). UCHIME improves sensitivity and speed of chimera detection. Bioinformatics 27, 2194–2200. 10.1093/bioinformatics/btr381 21700674 PMC3150044

[B18] Ellermann M. Gharaibeh R. Z. Fulbright L. Dogan B. Moore L. N. Broberg C. A. (2019). Yersiniabactin-Producing Adherent/Invasive Escherichia coli Promotes Inflammation-Associated Fibrosis in Gnotobiotic Il10(-/-). Mice Infect. Immun. 87 (11), e00587–19. 10.1128/IAI.00587-19 PMC6803345 31481410

[B19] Fei N. Zhao L. (2013). An opportunistic pathogen isolated from the gut of an obese human causes obesity in germfree mice. ISME J. 7, 880–884. 10.1038/ismej.2012.153 23235292 PMC3603399

[B20] Fouts D. E. Torralba M. Nelson K. E. Brenner D. A. Schnabl B. (2012). Bacterial translocation and changes in the intestinal microbiome in mouse models of liver disease. J. Hepatol. 56, 1283–1292. 10.1016/j.jhep.2012.01.019 22326468 PMC3357486

[B21] Gadd V. L. Skoien R. Powell E. E. Fagan K. J. Winterford C. Horsfall L. (2014). The portal inflammatory infiltrate and ductular reaction in human nonalcoholic fatty liver disease. Hepatology 59, 1393–1405. 10.1002/hep.26937 24254368

[B22] Gardner S. N. Slezak T. Hall B. G. (2015). kSNP3.0: SNP detection and phylogenetic analysis of genomes without genome alignment or reference genome. Bioinformatics 31, 2877–2878. 10.1093/bioinformatics/btv271 25913206

[B23] Himes R. W. Smith C. W. (2010). Tlr2 is critical for diet-induced metabolic syndrome in a murine model. FASEB J. 24, 731–739. 10.1096/fj.09-141929 19841034 PMC2830137

[B24] Itoh M. Kato H. Suganami T. Konuma K. Marumoto Y. Terai S. (2013). Hepatic crown-like structure: a unique histological feature in non-alcoholic steatohepatitis in mice and humans. PLoS One 8, e82163. 10.1371/journal.pone.0082163 24349208 PMC3859576

[B25] Jenne C. N. Kubes P. (2013). Immune surveillance by the liver. Nat. Immunol. 14, 996–1006. 10.1038/ni.2691 24048121

[B26] Jiang W. Wu N. Wang X. Chi Y. Zhang Y. Qiu X. (2015). Dysbiosis gut microbiota associated with inflammation and impaired mucosal immune function in intestine of humans with non-alcoholic fatty liver disease. Sci. Rep. 5, 8096. 10.1038/srep08096 25644696 PMC4314632

[B27] Jiao N. Baker S. S. Chapa-Rodriguez A. Liu W. Nugent C. A. Tsompana M. (2018). Suppressed hepatic bile acid signalling despite elevated production of primary and secondary bile acids in NAFLD. Gut 67, 1881–1891. 10.1136/gutjnl-2017-314307 28774887

[B28] Jun X. Ning C. Yang S. Zhe W. Na W. Yifan Z. (2019). Alteration of Fungal Microbiota After 5-ASA Treatment in UC Patients. Inflammation Bowel Dis. 26, 380–390. 10.1093/ibd/izz207 31750918

[B29] Kanehisa M. Goto S. Sato Y. Kawashima M. Furumichi M. Tanabe M. (2014). Data, information, knowledge and principle: back to metabolism in KEGG. Nucleic Acids Res. 42, D199–D205. 10.1093/nar/gkt1076 24214961 PMC3965122

[B30] Kaper J. B. Nataro J. P. Mobley H. L. (2004). Pathogenic Escherichia coli. Nat. Rev. Microbiol. 2, 123–140. 10.1038/nrmicro818 15040260

[B31] Kapil S. Duseja A. Sharma B. K. Singla B. Chakraborti A. Das A. (2016). Small intestinal bacterial overgrowth and toll-like receptor signaling in patients with non-alcoholic fatty liver disease. J. Gastroenterol. Hepatol. 31, 213–221. 10.1111/jgh.13058 26212089

[B32] Kawano Y. Cohen D. E. (2013). Mechanisms of hepatic triglyceride accumulation in non-alcoholic fatty liver disease. J. Gastroenterol. 48, 434–441. 10.1007/s00535-013-0758-5 23397118 PMC3633701

[B33] Kim H. N. Joo E. J. Cheong H. S. Kim Y. Kim H. L. Shin H. (2019). Gut Microbiota and Risk of Persistent Nonalcoholic Fatty Liver Diseases. J. Clin. Med. 8, 1089. 10.3390/jcm8081089 PMC6722749 31344854

[B34] Kolodziejczyk A. A. Zheng D. Shibolet O. Elinav E. (2019). The role of the microbiome in NAFLD and NASH. EMBO Mol. Med. 11 (2), e9302. 10.15252/emmm.201809302 30591521 PMC6365925

[B35] Kostic A. D. Chun E. Robertson L. Glickman J. N. Gallini C. A. Michaud M. (2013). Fusobacterium nucleatum potentiates intestinal tumorigenesis and modulates the tumor-immune microenvironment. Cell Host Microbe 14, 207–215. 10.1016/j.chom.2013.07.007 23954159 PMC3772512

[B36] Le Roy T. Llopis M. Lepage P. Bruneau A. Rabot S. Bevilacqua C. (2013). Intestinal microbiota determines development of non-alcoholic fatty liver disease in mice. Gut 62, 1787–1794. 10.1136/gutjnl-2012-303816 23197411

[B37] Ley R. E. Turnbaugh P. J. Klein S. Gordon J. I. (2006). Microbial ecology: human gut microbes associated with obesity. Nature 444, 1022–1023. 10.1038/4441022a 17183309

[B38] Li F. Hao X. Chen Y. Bai L. Gao X. Lian Z. (2017). The microbiota maintain homeostasis of liver-resident gammadeltaT-17 cells in a lipid antigen/CD1d-dependent manner. Nat. Commun. 7, 13839. 10.1038/ncomms13839 28067223 PMC5227332

[B39] Liao L. Schneider K. M. Galvez E. J. C. Frissen M. Marschall H. U. Su H. (2019). Intestinal dysbiosis augments liver disease progression via NLRP3 in a murine model of primary sclerosing cholangitis. Gut 68, 1477–1492. 10.1136/gutjnl-2018-316670 30872395

[B40] Liu R. Li X. Huang Z. Zhao D. Ganesh B. S. Lai G. (2018). C/EBP homologous protein-induced loss of intestinal epithelial stemness contributes to bile duct ligation-induced cholestatic liver injury in mice. Hepatology 67, 1441–1457. 10.1002/hep.29540 28926118 PMC5859257

[B41] Lotowska J. M. Sobaniec-Lotowska M. E. Lebensztejn D. M. (2013). The role of Kupffer cells in the morphogenesis of nonalcoholic steatohepatitis - ultrastructural findings. First Rep. Pediatr. Patients Scand. J. Gastroenterol. 48, 352–357. 10.3109/00365521.2012.746390 23268566

[B42] Lowe T. M. Eddy S. R. (1997). tRNAscan-SE: a program for improved detection of transfer RNA genes in genomic sequence. Nucleic Acids Res. 25, 955–964. 10.1093/nar/25.5.955 9023104 PMC146525

[B43] Magoc T. Salzberg S. L. (2011). FLASH: fast length adjustment of short reads to improve genome assemblies. Bioinformatics 27, 2957–2963. 10.1093/bioinformatics/btr507 21903629 PMC3198573

[B44] Manfredo Vieira S. Hiltensperger M. Kumar V. Zegarra-Ruiz D. Dehner C. Khan N. (2018). Translocation of a gut pathobiont drives autoimmunity in mice and humans. Science 359, 1156–1161. 10.1126/science.aar7201 29590047 PMC5959731

[B45] Miura K. Yang L. van Rooijen N. Brenner D. A. Ohnishi H. Seki E. (2013). Toll-like receptor 2 and palmitic acid cooperatively contribute to the development of nonalcoholic steatohepatitis through inflammasome activation in mice. Hepatology 57, 577–589. 10.1002/hep.26081 22987396 PMC3566276

[B46] Mouries J. Brescia P. Silvestri A. Spadoni I. Sorribas M. Wiest R. (2019). Microbiota-driven gut vascular barrier disruption is a prerequisite for non-alcoholic steatohepatitis development. J. Hepatol. (6), 1216–1228. 10.1016/j.jhep.2019.08.005 PMC6880766 31419514

[B47] Mouzaki M. Comelli E. M. Arendt B. M. Bonengel J. Fung S. K. Fischer S. E. (2013). Intestinal microbiota in patients with nonalcoholic fatty liver disease. Hepatology 58, 120–127. 10.1002/hep.26319 23401313

[B48] Mridha A. R. Haczeyni F. Yeh M. M. Haigh W. G. Ioannou G. N. Barn V. (2017). TLR9 is up-regulated in human and murine NASH: pivotal role in inflammatory recruitment and cell survival. Clin. Sci. (Lond) 131, 2145–2159. 10.1042/CS20160838 28687713

[B49] Peterson L. W. Artis D. (2014). Intestinal epithelial cells: regulators of barrier function and immune homeostasis. Nat. Rev. Immunol. 14, 141–153. 10.1038/nri3608 24566914

[B50] Roh Y. S. Seki E. (2013). Toll-like receptors in alcoholic liver disease, non-alcoholic steatohepatitis and carcinogenesis. J. Gastroenterol. Hepatol. 28 Suppl 1, 38–42. 10.1111/jgh.12019 PMC3721430 23855294

[B51] Safari Z. Gerard P. (2019). The links between the gut microbiome and non-alcoholic fatty liver disease (NAFLD). Cell Mol. Life Sci. 76, 1541–1558. 10.1007/s00018-019-03011-w 30683985 PMC11105223

[B52] Schnabl B. Brenner D. A. (2014). Interactions between the intestinal microbiome and liver diseases. Gastroenterology 146, 1513–1524. 10.1053/j.gastro.2014.01.020 24440671 PMC3996054

[B53] Schneider K. M. Mohs A. Kilic K. Candels L. S. Elfers C. Bennek E. (2019). Intestinal Microbiota Protects against MCD Diet-Induced Steatohepatitis. Int. J. Mol. Sci. 20 (2), 308. 10.3390/ijms20020308 PMC6358781 30646522

[B54] Schwimmer J. B. Johnson J. S. Angeles J. E. Behling C. Belt P. H. Borecki I. (2019). Microbiome Signatures Associated With Steatohepatitis and Moderate to Severe Fibrosis in Children With Nonalcoholic Fatty Liver Disease. Gastroenterology 157, 1109–1122. 10.1053/j.gastro.2019.06.028 31255652 PMC6756995

[B55] Sica A. Invernizzi P. Mantovani A. (2014). Macrophage plasticity and polarization in liver homeostasis and pathology. Hepatology 59, 2034–2042. 10.1002/hep.26754 24115204

[B56] Sorribas M. Jakob M. O. Yilmaz B. Li H. Stutz D. Noser Y. (2019). FXR-modulates the gut-vascular barrier by regulating the entry sites for bacterial translocation in experimental cirrhosis. J. Hepatol. (6), 1126–1140. 10.1016/j.jhep.2019.06.017 31295531

[B57] Spruss A. Kanuri G. Wagnerberger S. Haub S. Bischoff S. C. Bergheim I. (2009). Toll-like receptor 4 is involved in the development of fructose-induced hepatic steatosis in mice. Hepatology 50, 1094–1104. 10.1002/hep.23122 19637282

[B58] Tsoi H. Chu E. S. H. Zhang X. Sheng J. Nakatsu G. Ng S. C. (2017). Peptostreptococcus anaerobius Induces Intracellular Cholesterol Biosynthesis in Colon Cells to Induce Proliferation and Causes Dysplasia in Mice. Gastroenterology 152, 1419–1433.e5. 10.1053/j.gastro.2017.01.009 28126350

[B59] Vandanmagsar B. Youm Y. H. Ravussin A. Galgani J. E. Stadler K. Mynatt R. L. (2011). The NLRP3 inflammasome instigates obesity-induced inflammation and insulin resistance. Nat. Med. 17, 179–188. 10.1038/nm.2279 21217695 PMC3076025

[B60] Wang R. Li H. Yang X. Xue X. Deng L. Shen J. (2018). Genetically Obese Human Gut Microbiota Induces Liver Steatosis in Germ-Free Mice Fed on Normal Diet. Front. Microbiol. 9, 1602. 10.3389/fmicb.2018.01602 30079055 PMC6062601

[B61] Wiest R. Albillos A. Trauner M. Bajaj J. S. Jalan R. (2017). Targeting the gut-liver axis in liver disease. J. Hepatol. 67, 1084–1103. 10.1016/j.jhep.2017.05.007 28526488

[B62] Wu Z. Tan J. Chi Y. Zhang F. Xu J. Song Y. (2018). Mesenteric adipose tissue contributes to intestinal barrier integrity and protects against nonalcoholic fatty liver disease in mice. Am. J. Physiol. Gastrointest. Liver Physiol. 315, G659–G670. 10.1152/ajpgi.00079.2018 29902065

[B63] Xu J. Chen N. Wu Z. Song Y. Zhang Y. Wu N. (2018). 5-Aminosalicylic Acid Alters the Gut Bacterial Microbiota in Patients With Ulcerative Colitis. Front. Microbiol. 9, 1274. 10.3389/fmicb.2018.01274 29951050 PMC6008376

[B64] Yan H. Fei N. Wu G. Zhang C. Zhao L. Zhang M. (2016). Regulated Inflammation and Lipid Metabolism in Colon mRNA Expressions of Obese Germfree Mice Responding to Enterobacter cloacae B29 Combined with the High Fat Diet. Front. Microbiol. 7, 1786. 10.3389/fmicb.2016.01786 27877172 PMC5099522

[B65] Yu T. Guo F. Yu Y. Sun T. Ma D. Han J. (2017). Fusobacterium nucleatum Promotes Chemoresistance to Colorectal Cancer by Modulating Autophagy. Cell 170, 548–563.e16. 10.1016/j.cell.2017.07.008 28753429 PMC5767127

[B66] Yuan J. Chen C. Cui J. Lu J. Yan C. Wei X. (2019). Fatty Liver Disease Caused by High-Alcohol-Producing Klebsiella pneumoniae. Cell Metab. 30, 675–688.e7. 10.1016/j.cmet.2019.08.018 31543403

[B67] Zargar A. Quan D. N. Carter K. K. Guo M. Sintim H. O. Payne G. F. (2015). Bacterial secretions of nonpathogenic Escherichia coli elicit inflammatory pathways: a closer investigation of interkingdom signaling. mBio 6, e00025. 10.1128/mBio.00025-15 25759496 PMC4453519

[B68] Zhu L. Baker S. S. Gill C. Liu W. Alkhouri R. Baker R. D. (2013). Characterization of gut microbiomes in nonalcoholic steatohepatitis (NASH) patients: a connection between endogenous alcohol and NASH. Hepatology 57, 601–609. 10.1002/hep.26093 23055155

