# Supplementary figures and images for: E. coli NF73-1 Isolated From NASH Patients Aggravates NAFLD in Mice by Translocating Into the Liver and Stimulating M1 Polarization
Source: Front Cell Infect Microbiol. 2020 Dec 11;10:535940. doi: 10.3389/fcimb.2020.535940 (PMC7759485; doi:10.3389/fcimb.2020.535940)

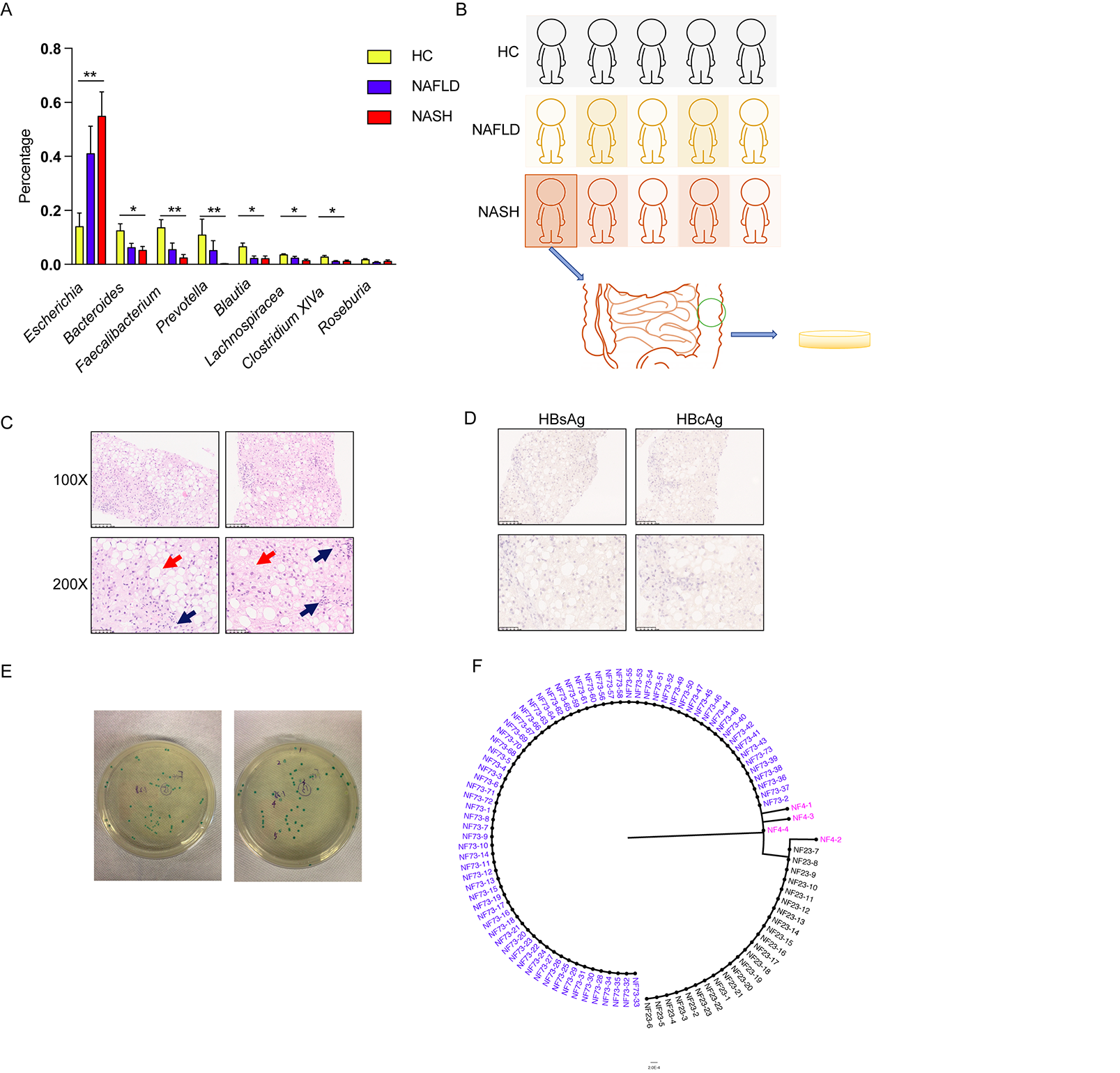

Supplement: Supplementary Figure 1 — Isolation and whole-genome sequencing of clinical Escherichia strains from the intestinal mucosa of NASH patients. (A) Comparison of the seven genera abundance among NASH and NAFLD patients and healthy subjects. (B) Schematic diagram of the overall design and bacterial isolation. (C) Two representative liver H&E images of NASH patients (blue arrows, inflammatory cells; red arrows, steatosis). (D) Negative immunostaining for HBsAg and HBcAg in the NASH patient. (E) Representative image of Escherichia strains on E. coli Chromogenic Medium. (F) Phylogenetic relationships among clinical E. coli strains inferred with the maximum likelihood method based on the full-length 16S rRNA sequence. The black scale bar indicates 100 μm and 50 μm. Data are calculated as the mean ± SEM, n = 9–10 per group. *p <0.05, **p <0.01 (one-way analysis of variance between multiple groups). [file Image_1.tif]

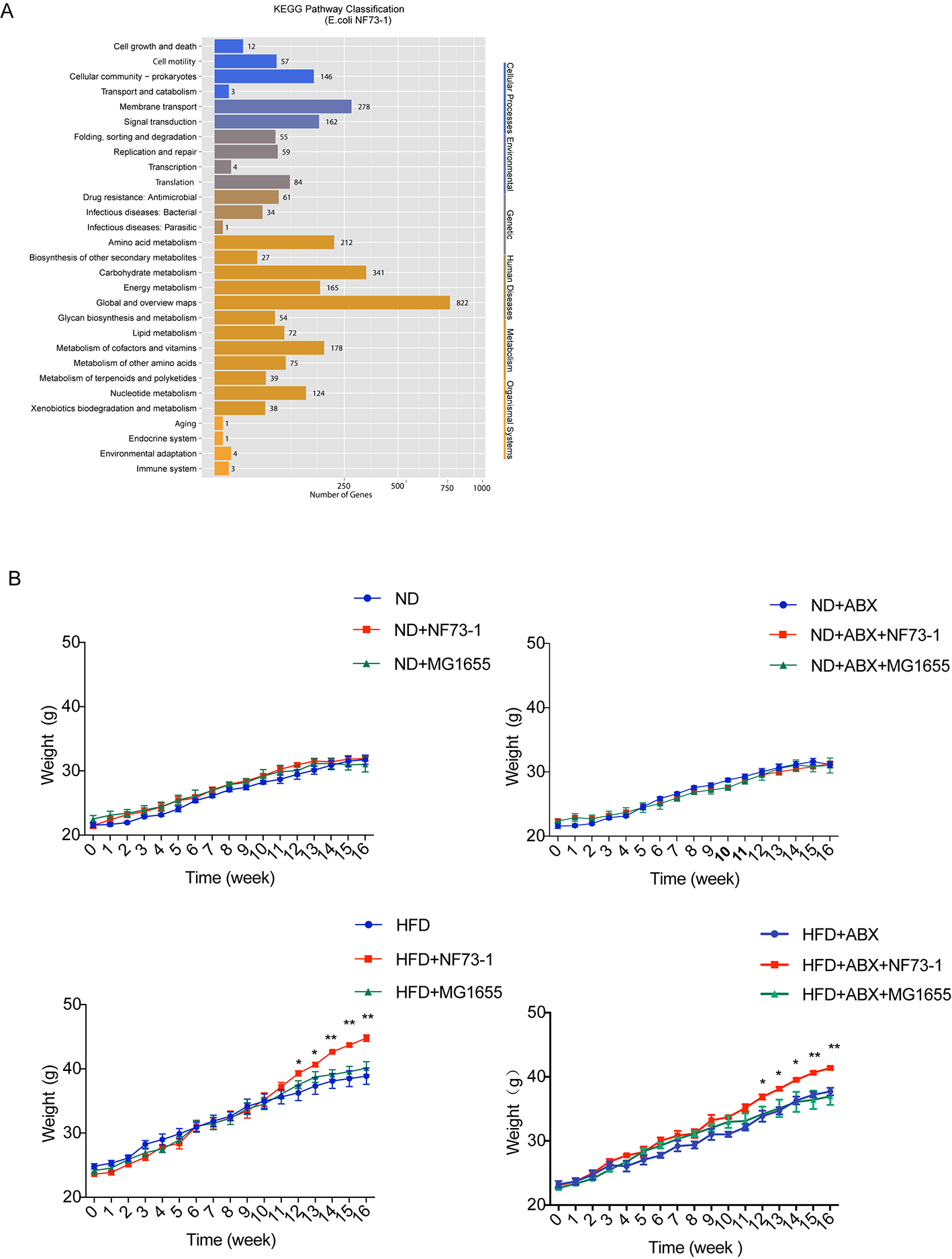

Supplement: Supplementary Figure 2 — General functional annotations of E. coli NF73-1 and body weight of mouse. (A) KEGG general functional annotations of E. coli NF73-1. (B) Body weight of mouse. Data are calculated as the mean ± SEM, n = 5 per group. *p <0.05, **p <0.01 (one-way analysis of variance between multiple groups). [file Image_2.tif]

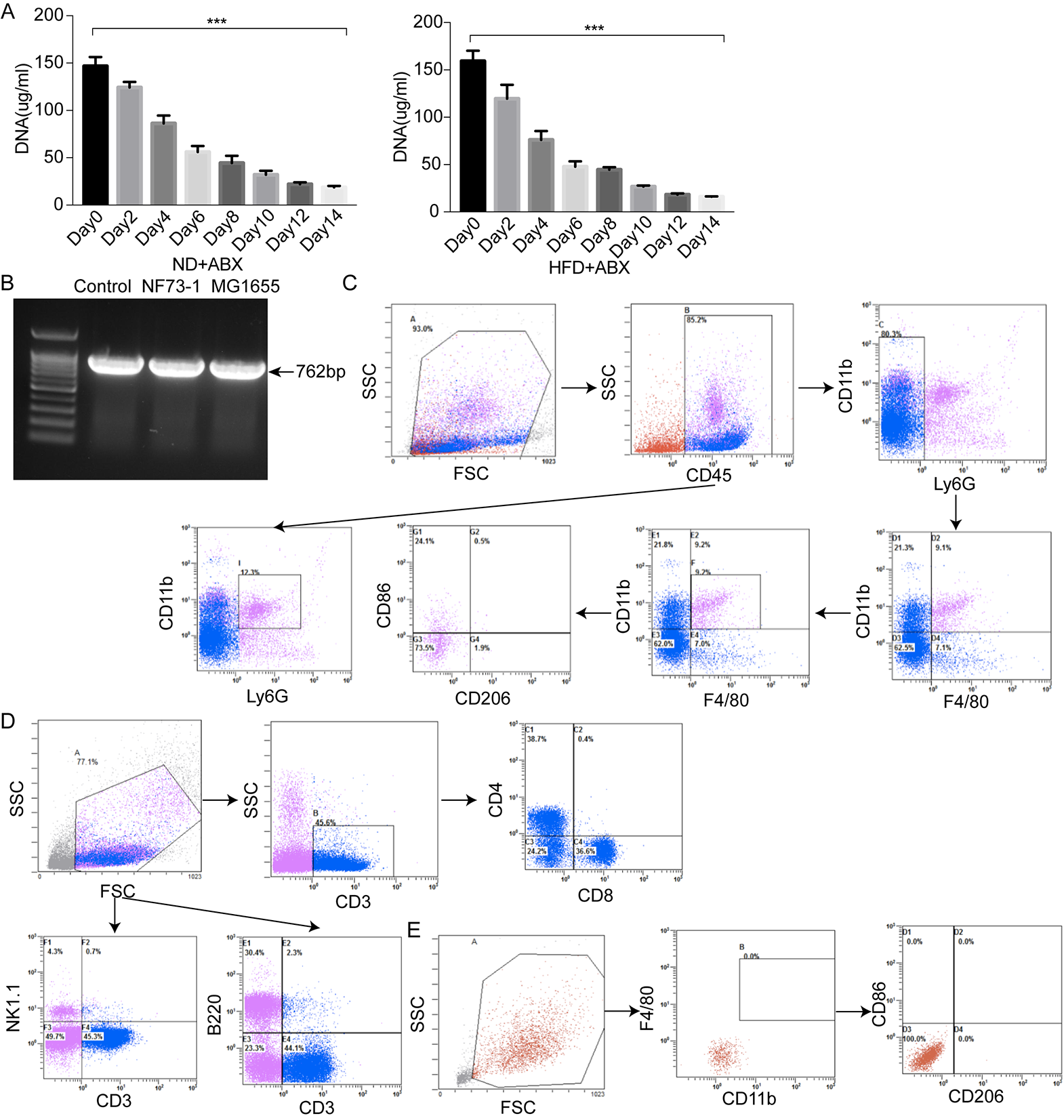

Supplement: Supplementary Figure 3 — Total bacteria in stool samples with administration of antibiotics in NAFLD mice and the generation of E. coli EGFP-NF73-1. (A) Total bacterial loads in stool samples of NAFLD mice and ND mice treated with antibiotics were determined from the DNA concentrations. Data are calculated as the mean ± SEM, n = 4–6 per group. **p <0.01 (one-way analysis of variance between multiple groups). (B) Representative image of EGFP expression of E. coli EGFP-NF73-1 and E. coli EGFP-MG 1655 strains and the positive control. (C) Flow cytometry gating strategies for liver macrophages. Representative flow cytometric plots. (D) Flow cytometry gating strategies for liver lymphocytes. Representative flow cytometric plots; (E) Flow cytometry gating strategies for RAW264.7 macrophages. Representative flow cytometric plots. [file Image_3.tif]

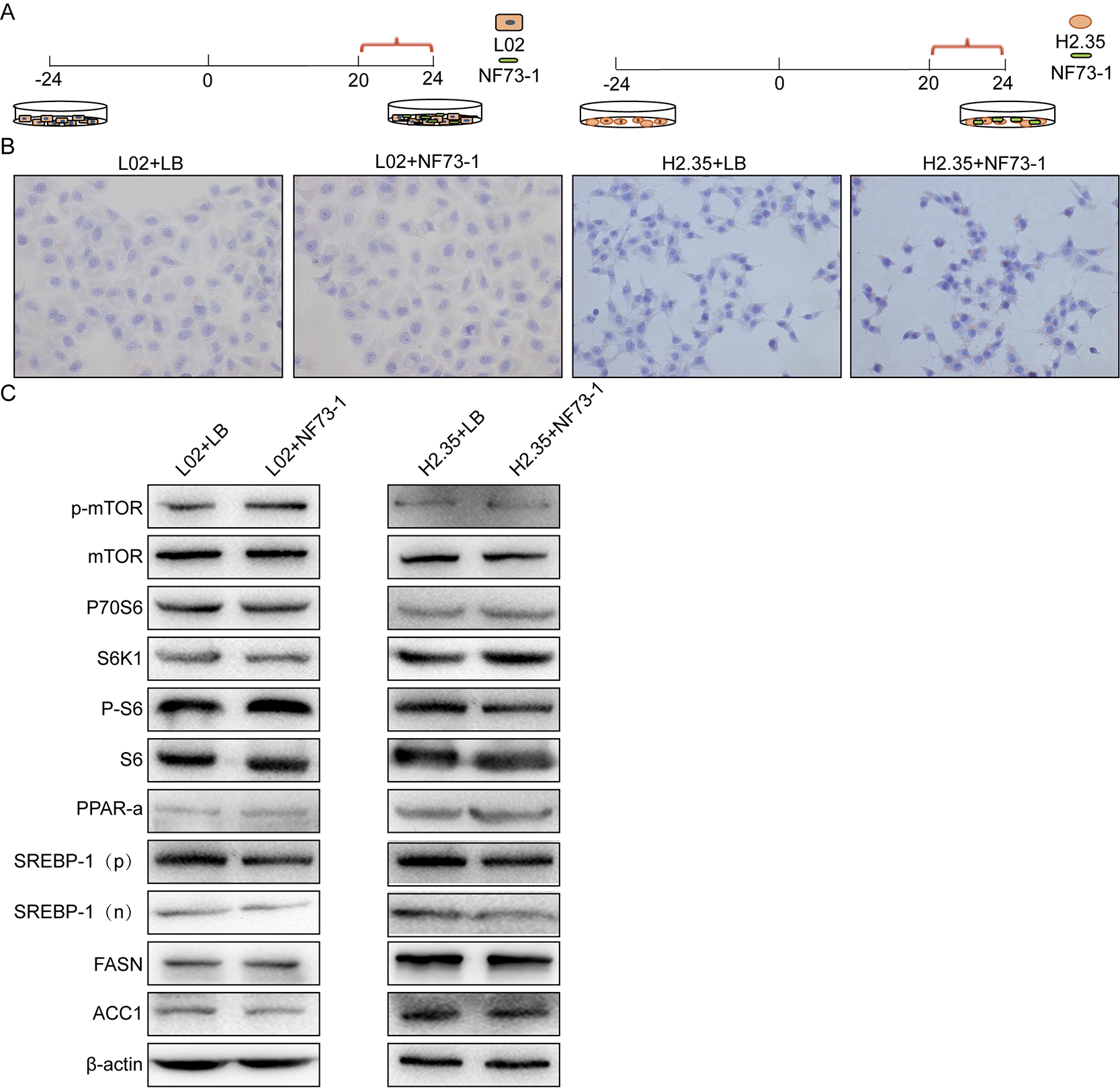

Supplement: Supplementary Figure 4 — E. coli NF73-1, the non-inducer of lipid accumulation in hepatocytes in vitro. (A) The human liver normal cell line L02 and mouse liver normal cell line H2.35 were exposed to E. coli NF73-1 (multiplicity of infection = 1:10) for 4 h. (B) Lipid accumulation determined by Oil Red O staining of palmitic acid- and oleic acid-treated hepatocytes cocultured with or without E. coli NF73-1. (C) Protein synthesis of phosphorylated mTOR, S6K1, S6, SREBP-1, ACC1, FASN, and PPAR-α in palmitic acid- and oleic acid-treated hepatocytes cocultured with or without E. coli NF73-1. mTOR, mammalian target of rapamycin; S6K1, S6 kinase 1; SREBP-1, sterol-regulatory element binding proteins-1; ACC1, acetyl-coenzyme A carboxylase; FASN, fatty acid synthase; PPAR-a, peroxisome proliferator-activated receptor alpha; N, cleaved nuclear (68 kDa) form of SREBP-1; P, precursor (125 kDa) form of SREBP-1. [file Image_4.tif]
